# Interaction between estrogen receptor-α and *PNPLA3* p.I148M variant drives fatty liver disease susceptibility in women

**DOI:** 10.1038/s41591-023-02553-8

**Published:** 2023-09-25

**Authors:** Alessandro Cherubini, Mahnoosh Ostadreza, Oveis Jamialahmadi, Serena Pelusi, Eniada Rrapaj, Elia Casirati, Giulia Passignani, Marjan Norouziesfahani, Elena Sinopoli, Guido Baselli, Clara Meda, Paola Dongiovanni, Daniele Dondossola, Neil Youngson, Aikaterini Tourna, Shilpa Chokshi, Elisabetta Bugianesi, EPIDEMIC Study Investigators, Luisa Ronzoni, Cristiana Bianco, Laura Cerami, Veronica Torcianti, Giulia Periti, Sara Margarita, Rossana Carpani, Francesco Malvestiti, Ilaria Marini, Melissa Tomasi, Angela Lombardi, Jessica Rondena, Marco Maggioni, Roberta D’Ambrosio, Valentina Vaira, Anna Ludovica Fracanzani, Chiara Rosso, Grazia Pennisi, Salvatore Petta, Antonio Liguori, Luca Miele, Federica Tavaglione, Umberto Vespasiani-Gentilucci, Marcello Dallio, Alessandro Federico, Giorgio Soardo, Jussi Pihlajamäki, Ville Männistö, Sara Della Torre, Daniele Prati, Stefano Romeo, Luca Valenti

**Affiliations:** 1 Precision Medicine—Biological Resource Center and Department of Transfusion Medicine, Fondazione IRCCS Ca’ Granda Ospedale Maggiore Policlinico, Milan, Italy; 2 Department of Molecular and Clinical Medicine, Gothenburg University, Gothenburg, Sweden; 3 Department of Pathophysiology and Transplantation, Università degli Studi di Milano, Milan, Italy; 4 Department of Health Sciences, Università degli Studi di Milano, Milan, Italy; 5 Medicine and Metabolic Diseases, Fondazione IRCCS Ca’ Granda Ospedale Maggiore Policlinico, Milan, Italy; 6 General and Liver Transplant Surgery, Fondazione IRCCS Ca’ Granda, Ospedale Maggiore Policlinico and University of Milan, Centre of Preclinical Research, Milan, Italy; 7 Foundation for Liver Research, The Roger Williams Institute of Hepatology, London, UK; 8 Faculty of Life Sciences and Medicine, King’s College London, London, UK; 9 Department of Medical Sciences, Division of Gastroenterology, University of Turin, Turin, Italy; 10 Department of Pharmaceutical Sciences, Università degli Studi di Milano, Milan, Italy; 11 Cardiology Department, Sahlgrenska Hospital, Gothenburg, Sweden; 12 Department of Medical and Surgical Science, Magna Græcia University, Catanzaro, Italy; 13 Department of Pathology, Fondazione IRCCS Ca’ Granda Ospedale Maggiore Policlinico, Milan, Italy; 14 Division of Gastroenterology and Hepatology Foundation, IRCCS Ca’ Granda Ospedale Maggiore Policlinico, Milan, Italy; 15 Department of Health Promotion, Mother and Child Care, Internal Medicine and Medical specialties (ProMISE), University of Palermo, Palermo, Italy; 16 Dipartimento Universitario Medicina e Chirurgia Traslazionale, Università Cattolica del Sacro Cuore, Rome, Italy; 17 Area Medicina Interna, Gastroenterologia e Oncologia Medica, Fondazione Policlinico A. Gemelli IRCCS, Rome, Italy; 18 Department of Internal Medicine, Research Unit of Hepatology, Università Campus Bio-Medico di Roma, Rome, Italy; 19 Department of Precision Medicine, University of Campania ‘Luigi Vanvitelli’, Naples, Italy; 20 Clinica Medica, Department of Medicine, European Excellence Center for Arterial Hypertension, University of Udine, Udine, Italy; 21 Department of Clinical Nutrition, Institute of Public Health and Clinical Nutrition, University of Eastern Finland, Kuopio, Finland; 22 Department of Medicine, Endocrinology and Clinical Nutrition, Kuopio University Hospital, Kuopio, Finland; 23 Department of Medicine, University of Eastern Finland and Kuopio, University Hospital, Kuopio, Finland

**Keywords:** Liver diseases, Personalized medicine

## Abstract

Fatty liver disease (FLD) caused by metabolic dysfunction is the leading cause of liver disease and the prevalence is rising, especially in women. Although during reproductive age women are protected against FLD, for still unknown and understudied reasons some develop rapidly progressive disease at the menopause. The patatin-like phospholipase domain-containing 3 (*PNPLA3*) p.I148M variant accounts for the largest fraction of inherited FLD variability. In the present study, we show that there is a specific multiplicative interaction between female sex and *PNPLA3* p.I148M in determining FLD in at-risk individuals (steatosis and fibrosis, *P* < 10^−10^; advanced fibrosis/hepatocellular carcinoma, *P* = 0.034) and in the general population (*P* < 10^−7^ for alanine transaminase levels). In individuals with obesity, hepatic *PNPLA3* expression was higher in women than in men (*P* = 0.007) and in mice correlated with estrogen levels. In human hepatocytes and liver organoids, *PNPLA3* was induced by estrogen receptor-α (ER-α) agonists. By chromatin immunoprecipitation and luciferase assays, we identified and characterized an ER-α-binding site within a *PNPLA3* enhancer and demonstrated via CRISPR–Cas9 genome editing that this sequence drives *PNPLA3* p.I148M upregulation, leading to lipid droplet accumulation and fibrogenesis in three-dimensional multilineage spheroids with stellate cells. These data suggest that a functional interaction between ER-α and *PNPLA3* p.I148M variant contributes to FLD in women.

## Abstract (web summary)

Analysis using a combination of molecular and genetic epidemiological approaches reveals an interaction between female sex and the genetic variant *PNPLA3* p.I148M that might explain why some women have increased susceptibility to fatty liver disease after onset of menopause.

## Main

FLD related to metabolic dysfunction, excessive alcohol intake and other hepatotoxic factors is the leading cause of liver disease worldwide^1–3^. FLD encompasses a wide spectrum of liver pathologies, ranging from intracellular accumulation of triglycerides to severe lipotoxicity leading to fibrosing steatohepatitis^4,5^, and is becoming the leading cause of liver transplantation, hepatocellular carcinoma (HCC) and liver-related mortality^2,3^. FLD is a heterogeneous condition triggered by sedentary lifestyle and excessive caloric intake, refined foods and hepatotoxins. These environmental factors synergize with genetic predisposition and epigenetic modifiers to induce liver disease^6^. Premenopausal women are protected against FLD, due to the beneficial impact of estrogens, acting on lipid metabolism at a systemic level and in hepatocytes mainly through the ER-α^7–10^. However, a fraction of women have increased susceptibility to development of FLD and HCC^11^, which can show a rapidly progressive course especially during and after the menopause when the protection conferred by high levels of estrogen is lost^12^. Nonalcoholic FLD is a rising cause of cirrhosis and HCC, disproportionately more in women than in men, especially in older individuals^13^. Furthermore, modulation of ER-α activity, for example, by tamoxifen, a selective ER modulator (SERM) used to treat ER-positive breast cancers, may even increase the risk of steatohepatitis in susceptible women^14^. However, FLD determinants in women are poorly understood and are understudied at the clinical, genetic and experimental levels^15^.

FLD has a large heritable component^16,17^ and the rs738409 C>G SNP of *PNPLA3* encoding the p.I148M variant is the most replicated genetic risk variant accounting for the largest proportion of FLD heritability^17–20^. Furthermore, the p.I148M genotype accounts for 16% and 27% of cirrhosis and HCC variability, respectively, in Europeans^16,17^. The mechanism underpinning the genetic association is still a matter of debate^21^, but it seems to require, both in vitro and in vivo, the presence of a p.I148M protein variant lacking enzymatic activity at the surface of intracellular lipid droplets in hepatocytes, where it competes with its homologous protein PNPLA2, reducing lipid droplet remodeling^22^. Consistently, in female mice engineered to carry *PNPLA3* p.I148M in hepatocytes, the mutant protein is resistant to ubiquitylation and proteasomal degradation, accumulating on lipid droplets and leading to steatosis development^23^. Furthermore, in hepatic stellate cells (HSCs), the *PNPLA3* p.I148M variant impairs the hydrolysis of retinyl esters, reducing extracellular retinol release, which leads to pro-fibrotic activity of HSCs^24–26^. The penetrance of the p.I148M variant on liver disease is amplified by adiposity and/or excess alcohol intake^6,27^. Some clues suggested that the *PNPLA3* p.I148M variant may have a larger impact in women than in men, as highlighted by a meta-regression of a meta-analysis of initial studies^28^, and the selective phenotypic expression in female knock-in mice on a sucrose diet^29,30^, but evidence remains circumstantial and the mechanism unclear.

The aim of the present study was therefore to test the interaction between the *PNPLA3* p.I148M variant and female sex in determining the predisposition to develop nonalcoholic–metabolic dysfunction-associated FLD, and to investigate the underlying mechanism.

## Results

### Interaction between *PNPLA3* p.I148M and female sex in determining FLD susceptibility

**Fig. 1:**
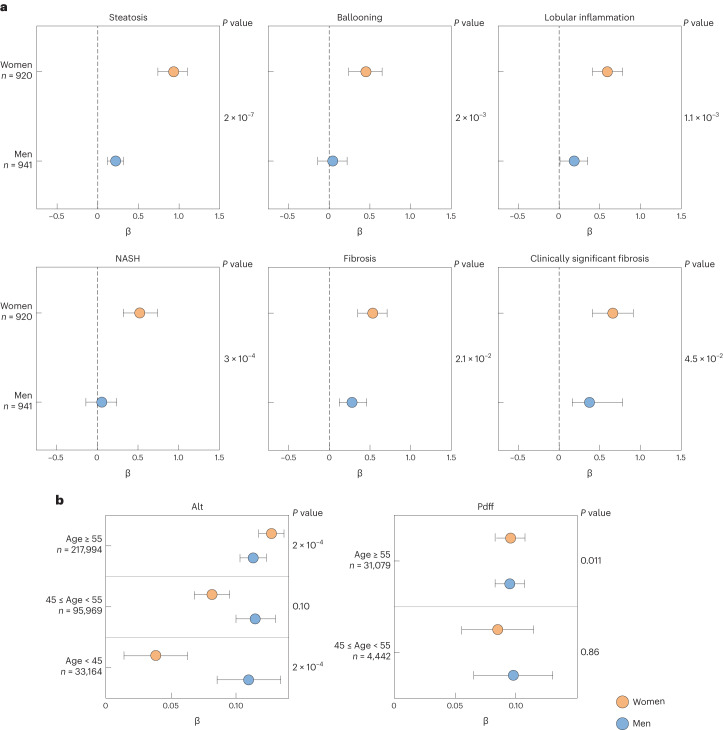
Impact of *PNPLA3* p.I148M variant on FLD susceptibility in women and men. **a**, Forest plot of association (estimates ± 95% confidence interval (CI)) between *PNPLA3* p.I148M variant and histological features of FLD in patients included in the Liver Biopsy Cohort stratified by sex (*n* = 1,861). **b**, Forest plot of association (estimates ± 95% CI) between *PNPLA3* p.I148M variant with ALT levels and hepatic fat concentration as measured by MRI–PDFF in the UK Biobank cohort (*n* = 347,127), after further stratification for age (<45 years: premenopausal, 45–55 years: perimenopausal, ≥55 years: postmenopausal). The impact of the variant was estimated using generalized linear regression models, under an additive genetic model for the *PNPLA3* variant, and were adjusted for age, BMI, T2D and recruitment modality. *P* values refer to the *PNPLA3* p.I148M × sex interaction term (Table 1).

We started from the observation that, in 1,861 European individuals who underwent histological assessment of liver damage for suspected nonalcoholic steatohepatitis (NASH), namely the Liver Biopsy Cohort^31^, the effect size of the *PNPLA3* p.I148M variant was larger in women than in men (Fig. 1a and Table 1). The impact of the p.I148M variant on histological and biochemical features of liver damage in the Liver Biopsy Cohort is shown in Supplementary Table 1 (upper panel). Although women were generally protected against FLD (*P* = 2 × 10^−6^ for protection against steatosis; *P* < 0.005 for all histological features), carriage of the p.I148M variant conferred a larger increase in the risk of the entire spectrum of FLD in women than in men (*P* = 2 × 10^−21^ for steatosis, *P* < 10^−4^ for all other histological features). There was a multiplicative interaction between female sex and the p.I148M variant on all liver damage outcomes (right column), resulting in a larger relative risk conferred in women compared with men (*P* = 2 × 10^−7^ for steatosis; *P* < 0.05 for all; Fig. 1a).

We next stratified patients by age to capture the approximated reproductive stage in women and examined the impact on steatosis grade as the FLD hallmark (Supplementary Table 2). Generally, women aged <45 years (premenopausal) were protected against steatosis, but this protection was lost in women aged approximately 45–54 years (perimenopausal) and women aged ≥55 years (postmenopausal). There was a multiplicative interaction between the p.I148M variant and female sex in determining steatosis in all groups, with a larger contribution of the *PNPLA3* × sex interaction in women aged ≥55 years. In contrast, despite the other major FLD risk, variants in *TM6SF2* and *MBOAT7* were associated with NASH and fibrosis, and there was no significant interaction of these variants with female sex on any feature of liver damage, with a very similar effect size in men and women (Supplementary Table 3).

In an independent case–control cohort of severe metabolic FLD (*n* = 4374)^32^, we confirmed the presence of an interaction between female sex and *PNPLA3* p.I148M variant in determining the risk of severe FLD progressing to advanced fibrosis and/or HCC (*P* < 0.05; Supplementary Table 1, middle panel). Consistently, the interaction with female sex was specific for the *PNPLA3* variant and not present for other FLD genetic risk factors (Supplementary Table 3). As a second independent replication, in a cohort of otherwise healthy individuals with metabolic dysfunction at high risk of FLD from the Liver-Bible-2022 cohort (*n* = 1,142)^33^, we observed an interaction between the p.I148M variant and female sex in determining alanine transaminase (ALT) levels, a biomarker of FLD and hepatic inflammation^16^ (+0.051 ± 0.02, *P* = 0.031; Supplementary Table 1). The effect was more marked (+0.068 ± 0.034, *P* = 0.047) in participants aged >55 years, consistent with postmenopausal status in women (Supplementary Table 2).

**Extended Data Fig. 1:**
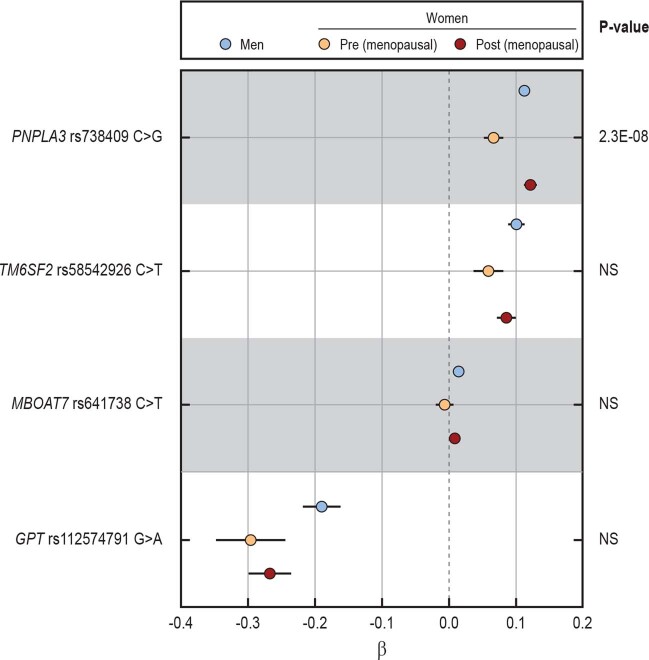
Comparison of the impact of PNPLA3 with other risk variants on ALT levels in UKBB Cohort stratified by sex and menopause. Forest plot of association (estimates ± 95% c.i.) between *PNPLA3* p.I148M, *TM6SF2* p.E167K, *MBOAT7-TMC4* rs641738 C > G and *GPT* p.R107K variants with sex on ALT levels in individuals in UKBB Cohort (n = 347,127) stratified by sex and menopause status. The impact of the variant was estimated by ordinal, logistic or generalized linear regression models, where appropriate under an additive genetic model for the different genetic variants, and were adjusted for age, BMI, T2D and recruitment modality. The impact of the variant was estimated by generalized linear regression models, under an additive genetic model for the *PNPLA3* variant, and were adjusted for age, BMI, T2D and recruitment modality.

**Extended Data Fig. 2:**
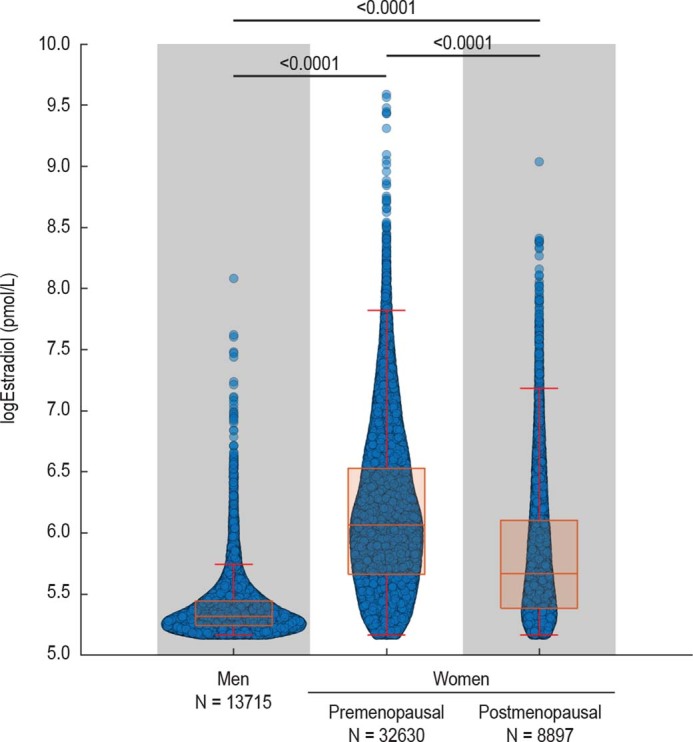
Impact of sex and menopause status on estradiol levels in UKBB Cohort. Violin plot showing estradiol levels in individuals in UKBB Cohort stratified by sex and menopause status. In the box and whisker plots, the line in the middle of the box represents the medians, tops and bottoms of the boxes represent the 25^th^ and 75^th^ quartiles respectively, and the whiskers showed the 5^th^ and 95^th^ percentile. The impact of sex and/or menopause status was estimated by unadjusted generalized linear regression models.

In the population-based UK Biobank cohort (*n* = 347,127)^34^, there was an age-dependent interaction between the *PNPLA3* p.I148M variant and female sex on ALT levels and hepatic fat content (Fig. 1b and Supplementary Tables 1 and 2). Indeed, there was an age-dependent detrimental effect of the variant on ALT with the lowest effect size in women aged <45 years and the largest effect size in women aged >55 years (Supplementary Table 2). Consistently, there was an interaction of *PNPLA3* p.I148M, female sex and menopause on ALT (0.047 ± 0.008, *P* = 2.3 × 10^−8^; Extended Data Fig. 1). Similarly, when looking at hepatic fat content (as determined by magnetic resonance imaging proton density fat fraction (MRI–PDFF)), there was an age-dependent interaction between female sex and the p.I148M variant, with a larger effect size observed in women aged >55 years (0.042 ± 0.017, *P* < 0.05; Fig. 1b and Supplementary Table 2). Although circulating levels of 17β-estradiol (E_2_) decreased in postmenopausal compared with premenopausal women (<45 years: premenopausal, ≥55 years: postmenopausal), they were still higher than in men (0.829 ± 0.011, *P* < 0.001; Extended Data Fig. 2).

**Extended Data Fig. 3:**
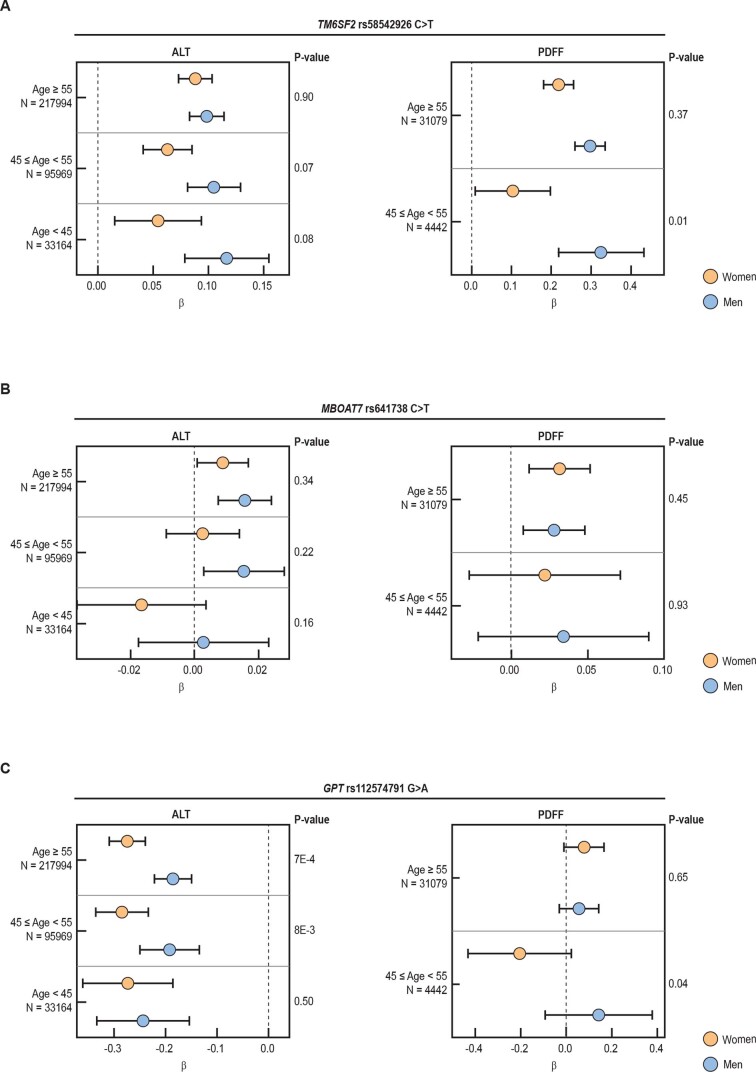
Impact of other genetic risk variants on ALT levels and hepatic fat in UKBB Cohort stratified for sex and age. Forest plot of association (estimates ± 95% c.i.) between *TM6SF2* p.E167K (Panel A), *MBOAT7* rs641738 (Panel B) and *GPT* p.R107K (Panel C) variants with sex on ALT and hepatic fat (MRI-PDFF) levels in individuals in UKBB Cohort (n = 347,127) after stratification for age (<45: pre-menopausal, 45-55: perimenopausal, ≥55 post-menopausal). The impact of the genetic variants was estimated by generalized linear regression models, under an additive genetic model, and were adjusted for age, BMI, T2D and recruitment modality. Source data

To verify whether the age-specific increase in the risk of liver damage was specific for the *PNPLA3* p.I148M variant, we examined *TM6SF2* p.E167K, *MBOAT7*-*TMC4* rs641738 C>T and *GPT* p.R107K, a direct modulator of the levels of the encoded ALT protein. The effect size of the risk alleles at these loci was consistently attenuated in women aged <45 years, possibly due to a reduced risk of liver damage in this subset of the population (−0.042 ± 0.024, −0.018 ± 0.013 and −0.036 ± 0.055, respectively; Extended Data Fig. 3), but they all consistently displayed a smaller effect on ALT and hepatic fat levels in women than in men irrespective of age.

All in all, these data demonstrate the existence of a multiplicative interaction between female sex and the *PNPLA3* p.I148M variant in determining all stages of FLD, becoming more evident after the age of 45 years (menopause) when the protection conferred by high fluctuating estrogen levels is lost in many women.

### Hepatic *PNPLA3* p.I148M expression, steatosis and fibrogenesis according to sex

**Fig. 2:**
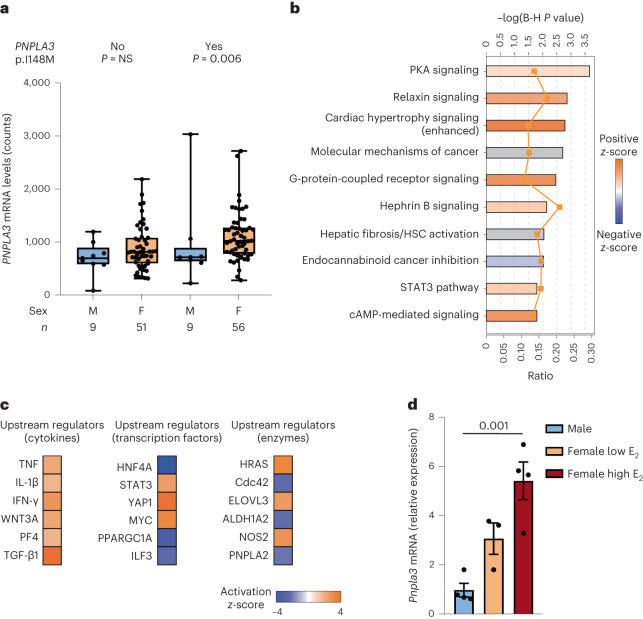
Estrogens regulate hepatic *PNPLA3* mRNA expression. **a**, Hepatic *PNPLA3* expression in patients in the transcriptomic cohort (*n* = 125 individuals with obesity) stratified by sex and carriage of the *PNPLA3* p.I148M variant. In the box and whisker plots, the line in the middle of the box represents the medians, tops and bottoms of the boxes the 25th and 75th quartiles, respectively, and the whiskers the minimum to maximum value. The impact of the variant was estimated using generalized linear regression models adjusted for age and batch. **b**, IPA of differentially regulated genes in women carrying the *PNPLA3* p.I148M variant. Terms are reported with a *P* < 0.05 after a Benjamini–Hochberg (B-H) correction. PKA, Protein kinase A. **c**, Predicted activation state of upstream cytokines, transcription factors and enzymes in women carrying the p.I148M variant. **d**, The mRNA levels of *Pnpla3* from RNA-seq analysis performed in the livers of male and female mice at low and high levels of E_2_. Data are presented as mean ± s.e.m. (*n* = 4 independent male and female high E_2_ mice; *n* = 3 independent female low E_2_ mice). One-way ANOVA was followed by Bonferroni’s post hoc test. Source data

**Extended Data Fig. 4:**
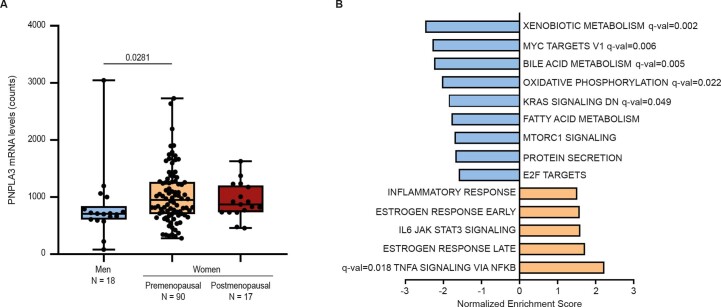
Additional information on PNPLA3 expression according to age and sex and PNPLA3 targetpathways in women from the Transcriptomic cohort. a) Hepatic *PNPLA3* expression in patients in the Transcriptomic cohort stratified by sex and menopause status. In the box and whisker plots, the line in the middle of the box represents the medians, tops and bottoms of the boxes represent the 25^th^ and 75^th^ quartiles respectively, and the whiskers showed the 5^th^ and 95^th^ percentile; Kruskal-Wallis followed by Dunn’s post hoc test. b) Gene set enrichment analysis (GSEA) of pathways enriched in genes differentially expressed in women carrying *PNPLA3* p.I148M variant; are considered only the terms with a p < 0.05. Source data

We next tested whether the heightened predisposition to FLD in women carrying the p.I148M variant may be mediated by modulation of hepatic *PNPLA3* expression. To this end, we examined hepatic *PNPLA3* expression in the transcriptomic cohort (*n* = 125) of individuals with obesity stratified by sex and *PNPLA3* genotype (Fig. 2a). We found that both carriage of the p.I148M variant and female sex were independently associated with higher hepatic *PNPLA3* messenger RNA expression (*P* = 0.002 and *P* = 0.007, respectively; Supplementary Table 4). Although *PNPLA3* mRNA levels in noncarriers showed no difference according to sex (*P* = 0.159), women carrying the p.I148M variant showed higher *PNPLA3* expression than men (*P* = 0.006). The different levels of circulating E_2_ observed in pre- and postmenopausal women and men were mirrored by the hepatic *PNPLA3* mRNA levels (Extended Data Fig. 4a). Consistent with *PNPLA3* being upregulated by the SREBP1C pathway^35^, in the subset of patients with available data (*n* = 40), fasting insulin was associated with higher *PNPLA3* levels (estimate 0.013 ± 0.006; *P* = 0.042).

We next identified at the transcriptome level a set of 1,619 genes that were differentially regulated by the *PNPLA3* p.I148M variant and female sex interaction (nominal *P* value < 0.05; Supplementary Data 1). These included genes involved in hepatic fibrogenesis, such as several collagen types (*COL3A1*, *COL4A1*, *COL4A2*, *COL4A4*, *COL5A1*, *COL5A3* and *COL6A6*), the TIMP (tissue inhibitors of metalloproteinases) metalloproteinase inhibitor 2 (*TIMP2*) and transforming growth factor-β1 (*TGFB1*), but also genes involved in lipid synthesis including fatty acyl-CoA reductases (Far) 1 (*FAR1*) and serine palmitoyltransferase long-chain base subunit 2 (*SPTLC2*). Ingenuity pathway analysis (IPA) and gene set enrichment analysis (GSEA) identified upregulation of pathways closely related to the development of liver fibrosis and inflammation, such as signal transducer and activator of transcription 3 (STAT3) and tumor necrosis factor-α (TNF-α)^36^. We also observed an induction of relaxin signaling that has been implicated in the sex-specific modulation of HSC activation^37^. On the other hand, pathways related to insulin signaling and lipogenesis (for example, mammalian target of rapamycin complex 1 signaling) were downregulated (Fig. 2b and Extended Data Fig. 4b). Consistent with this observation, IPA upstream regulator analysis predicted activation of classic inflammatory regulators transforming TGF-β1, TNF, interleukin-1β (IL-1β) and interferon-γ (IFN-γ), as well as transcription factors STAT3 and yes1-associated transcriptional regulator (YAP1) (Fig. 2c). Applying this approach to upstream enzymes additionally predicted inhibition of *PNPLA2*, supporting the reduction of remodeling of lipid droplets in women carrying the p.I148M variant (Fig. 2c).

To evaluate whether higher expression of *PNPLA3* in women may be dependent on sex hormones, we examined the effect of sex and the phase of estrous cycle on hepatic *Pnpla3* mRNA levels in C57/Bl6 mice^38^. We found that hepatic expression of *Pnpla3* was higher in female than in male mice and hepatic *Pnpla3* mRNA levels were higher during the follicular phase of the cycle characterized by high E_2_ levels than during the luteal phase characterized by lower E_2_ levels (4.445 ± 0.803, *P* < 0.01; Fig. 2d). Together, these results suggest that estrogens may be involved in upregulating PNPLA3 levels in the female liver.

### Effects of ER-α on *PNPLA3* expression and p.I148M variant phenotypic expression

**Fig. 3:**
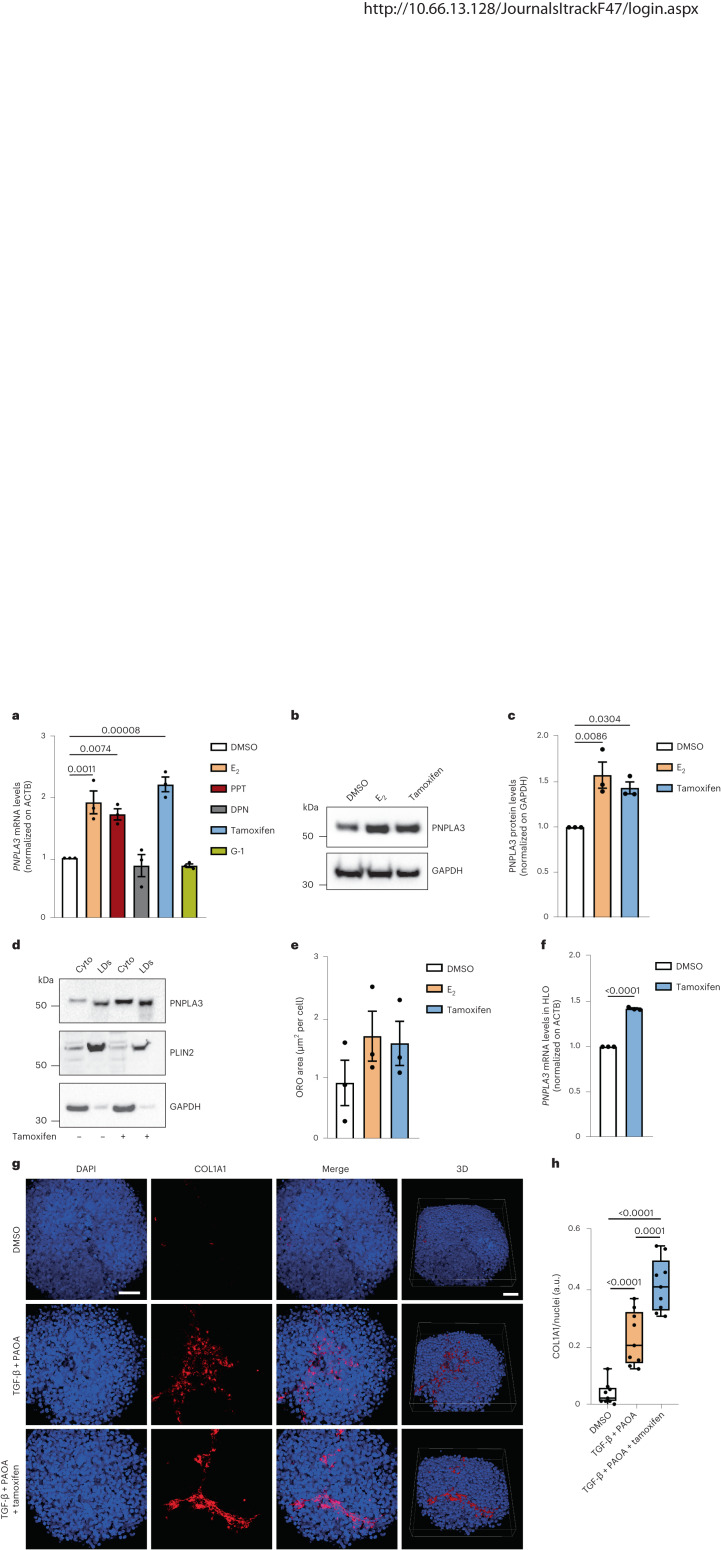
Effect of estrogen-induced *PNPLA3* expression on lipid droplet accumulation in human hepatocytes. **a**, RT-qPCR analysis of *PNPLA3* mRNA level in the human HepG2 cell line treated for 48 h with E_2_ (1 μM), PPT (1 μM), DPN (1 μM), tamoxifen (10 μM), G-1 (1 μM) and dimethyl sulfoxide (DMSO) as a negative control. **b**,**c**, Western blot analysis of PNPLA3 protein levels in HepG2 cells treated with E_2_ or tamoxifen and DMSO as a negative control (glyceraldehyde 3-phosphate dehydrogenase (GAPDH) used as loading control run on a different gel) (**b**) and relative quantification (**c**). **d**, Western blot analysis of PNPLA3, PLIN2 and GAPDH proteins in cytosolic and lipid droplet fractions obtained from cells treated for 48 h with fatty acids (oleic and palmitic acids, both at 250 μM) and tamoxifen or DMSO as a negative control. **e**, RT-qPCR analysis of *PNPLA3* mRNA level in HLOs treated for 48 h with tamoxifen (10 μM) and DMSO as a negative control. **f**, Relative quantification of ORO staining for visualization of intracellular neutral lipids of HepG2 treated for 48 h with E_2_ (1 μM), tamoxifen (10 μM) and DMSO as a negative control. **g**, Immunofluorescence staining of DAPI (blue) and COL1A1 (red) of 3D spheroids (HepG2:LX-2, 24:1) treated for 48 h with a mix of palmitic and oleic acids (PAOA, 250 μM each), TGF-β (10 ng ml^−1^) and tamoxifen (10 μM) or DMSO as a negative control. Scale bar, 50 μm. **h**, Quantification of COL1A1 levels by ImageJ normalized to DAPI quantification. a.u., arbitrary units. Whiskers show minimum to maximum values, box bounds the 25th to 75th percentiles and the center the median value. Data in **a**, **c** and **e** are presented as mean ± s.e.m. (*n* = 3 independent experiments). One-way ANOVA followed by Bonferroni’s post hoc test were used. Data in **f** are represented as mean ± s.e.m. (*n* = 3 independent experiments). A two-sided, unpaired Student’s *t*-test was used. Data in **h** are mean ± s.e.m. (*n* = 9 from 3 independent experiments). A one-way ANOVA was followed by Bonferroni’s post hoc test. Source data

Hepatocytes express three ERs: nuclear hormone receptor-α (ER-α) and -β (ER-β) and cell surface G-protein-coupled receptor (GPER), with ER-α being the most abundant^39^. To investigate whether ERs regulate *PNPLA3* expression in hepatocytes, human HepG2 hepatoma cells bearing the p.I148M variant in homozygosity^32,40^ were treated for 48 h with different ER modulators. *PNPLA3* mRNA expression levels were upregulated by incubation with E_2_ (*P* < 0.01; Fig. 3a), the main estrogen with ER-α/ER-β agonist activity, and by the selective ER-α agonist PPT (1,3,5-tris(4-hydroxyphenyl)-4-propyl-1*H*-pyrazole). In keeping with the estrogen effect observed above, the SERM tamoxifen, acting as ER-α agonist in the liver^41–43^, upregulated *PNPLA3* (Fig. 3a). On the other hand, the selective ER-β agonist DPN (2,3-bis(4-hydroxyphenyl)propionitrile) and the selective GPER agonist G-1 had no impact on *PNPLA3* mRNA levels (Fig. 3a). Incubation with E_2_ and tamoxifen resulted in higher PNPLA3 protein levels (Fig. 3b,c). These data suggest that ER-α agonists promote PNPLA3 synthesis in hepatocytes. Furthermore, exposure of HepG2 to tamoxifen for 48 h led to the selective accumulation of the variant PNPLA3 p.I148M protein lacking enzymatic activity on intracellular lipid droplets, thereby potentially hampering lipid droplet remodeling (Fig. 3d).

**Extended Data Fig. 5:**
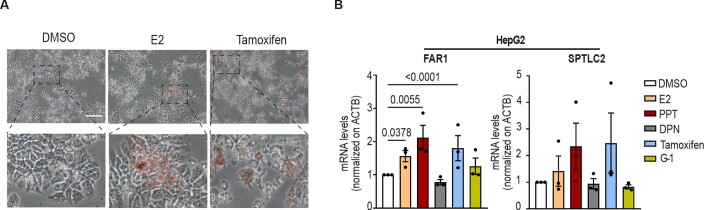
Impact of ERα agonists on intracellular lipids and lipotoxicity in HepG2 human hepatocytes. A) Representative images of Oil Red O (ORO) staining for visualization of intracellular neutral lipids of HepG2 treated for 48 h with E2 (1 μM), tamoxifen (10 μM) and DMSO as negative control. Scale bar, 200 μM. B) qRT-PCR analysis of FAR1 and SPTLC2 mRNA level in human HepG2 cell line treated for 24 h with E2 (1 μM), PPT (1 μM), DPN (1 μM), tamoxifen (10 μM), G-1 (1 μM) and DMSO as negative control. Data in panels B are presented as mean ± SEM (n = 3 independent experiments); one-way ANOVA followed by Bonferroni’s post hoc test. Source data

Consistent with the notion that the accumulation of p.I148M protein variant favors intracellular lipid retention, treatment with E_2_ and tamoxifen for 48 h increased the intracellular neutral lipid levels (Fig. 3e and Extended Data Fig. 5a). Next, we examined whether the expression of genes involved in lipotoxicity and liver damage upregulated by the sex *×* *PNPLA3* interaction in the transcriptomic cohort was also induced by E_2_ in HepG2 cells. We confirmed that, in the cells homozygous for the p.I148M, ER-α agonists upregulated *FAR1* and *SPTLC2* (Extended Data Fig. 5b).

**Extended Data Fig. 6:**
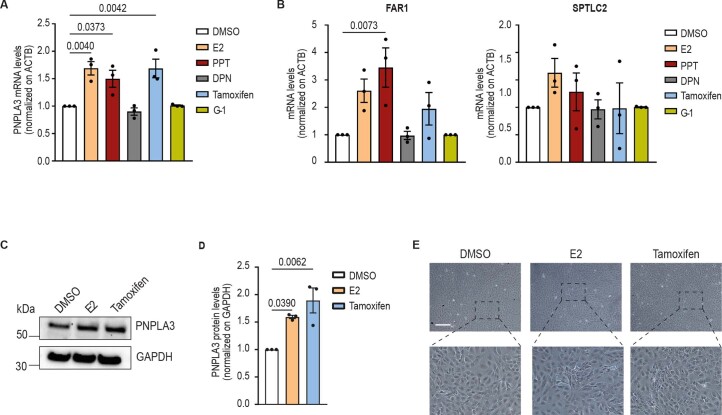
Impact of ERα agonists on PNPLA3 expression, intracellular lipids and lipotoxicity in HepaRG human hepatocytes. A) qRT-PCR analysis of *PNPLA3* mRNA level in human HepaRG cell line treated for 24 h with E2 (1 μM), PPT (1 μM), DPN (1 μM), tamoxifen (10 μM), G-1 (1 μM) and DMSO as negative control. B) qRT-PCR analysis of FAR1 and SPTLC2 mRNA level in human HepaRG cell line treated for 24 h with E2 (1 μM), PPT (1 μM), DPN (1 μM), tamoxifen (10 μM), G-1 (1 μM) and DMSO as negative control. C) Western blot analysis of PNPLA3 protein levels in HepG2 cells treated with E2 or tamoxifen and DMSO as negative control and D) relative quantification. E) Representative images of Oil Red O (ORO) staining for visualization of intracellular neutral lipids of HepaRG treated for 48 h with E2 (1 μM), tamoxifen (10 μM) and DMSO as negative control. Scale bar, 200 μM. Data in panel A, B and D are presented as mean ± SEM (n = 3 independent experiments); one-way ANOVA followed by Bonferroni’s post hoc test. Source data

We replicated the analyses in the HepaRG, a human female hepatoma cell line carrying wild-type *PNPLA3* (p.148I/I genotype). We observed that ER-α agonists upregulated *PNPLA3*, *FAR1* and *SPTLC2* mRNA expression levels (Extended Data Fig. 6a,b), as well as PNPLA3 at protein levels (Extended Data Fig. 6c,d), suggesting that estrogens upregulate PNPLA3 also in human female cells. However, consistent with wild-type PNPLA3 being a functional protein facilitating lipid remodeling, in HepaRG the increased synthesis of wild-type PNPLA3 did not result in an accumulation of lipid droplets (Extended Data Fig. 6e).

To confirm these results in primary cultures of human liver cells, we isolated hepatic progenitors from surgery samples, grew hepatic liver organoids (HLOs) and differentiated them toward hepatocytes according to a validated protocol^44^ (*n* = 3: 1 female, 2 males; 1 *PNPLA3* p.148I/M and 2 p.148 M/M). In this model, exposure to ER-α agonists for 48 h was confirmed to induce upregulation of *PNPLA3* mRNA levels (Fig. 3f; *P* < 0.05).

Finally, to test a causal role of ER-α-dependent induction of steatosis on fibrosis development, HepG2 cells were cocultured with human immortalized hepatic stellate cells (LX-2, homozygous for the p.I148M variant) to generate three-dimensional (3D) multilineage hepatic spheroids^45^. Spheroids treated for 48 h with TGF-β, fatty acids (palmitic and oleic acids) and tamoxifen showed increased collagen-I (COL1A1) synthesis and deposition compared with spheroids treated with TGF-β and fatty acids alone (Fig. 3g,h).

Taken together, these data indicate that ER-α-induced upregulation of PNPLA3 triggers lipid accumulation in hepatocytes promoting HSC activation and collagen deposition.

### *PNPLA3* induction via direct ER-α binding to an enhancer site

**Fig. 4:**
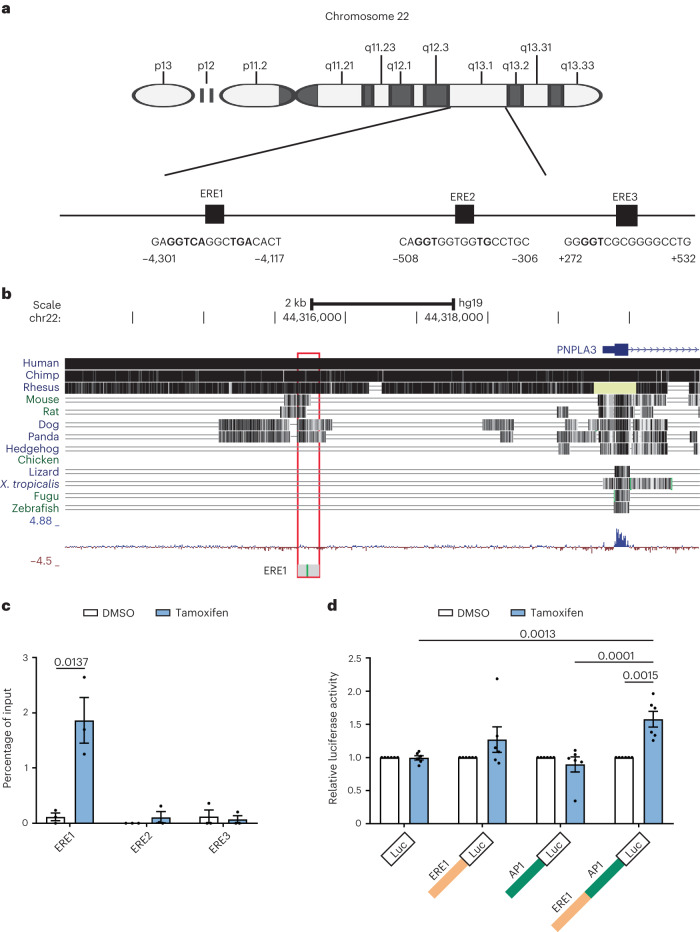
Prediction and identification of ER-α-binding sites at the promoter region of the *PNPLA3* gene. **a**, Representation of EREs located at the TSS and promoter region of the *PNPLA3* gene. The coordinates are based on the GRCh37/hg19 build (National Center for Biotechnology Information (NCBI) reference sequence NC_000022.10). **b**, PNPLA3-ERE1 showing a high degree of conservation across other mammal genomes. The coordinates are based on the GRCh37/hg19 build (NCBI reference sequence NC_000022.10). **c**, ChIP in HepG2 treated for 24 h with tamoxifen (10 μM) or DMSO as a negative control. The levels of ER-α at the ERE1, ERE2 and ERE3 of the *PNPLA3* gene were measured by RT-qPCR. Data are presented as mean ± s.e.m. (*n* = 3 independent experiments). The unpaired Student’s *t*-test was used. **d**, Luciferase (Luc) reporter activity analysis measuring the impact of tamoxifen on the transcriptional regulation of the *PNPLA3* putative enhancer region. The sequence containing both ERE1 and AP-1 sequences or ERE1 and AP-1 alone was cloned above the luciferase construct. Data in **c** are mean ± s.e.m. (*n* = 3 independent experiments). A two-sided, unpaired Student’s *t*-test was used. Data in **d** are mean ± s.e.m. (*n* = 6 independent experiments). A two-way ANOVA was used followed by Bonferroni’s multiple-comparison test. Source data

We next asked whether *PNPLA3* gene transcription is directly regulated by ER-α. At the upstream human *PNPLA3* promoter region, we identified three ER elements (EREs) (Fig. 4a). Among them, the first ERE sequence (from −4,303 to −4,119 before the transcription start site (TSS)), henceforth called PNPLA3-ERE1, was highly conserved among mammals (Fig. 4b), whereas it was completely lost in primitive vertebrates. These data are consistent with the presence of specific reproductive, immune and metabolic functions of estrogen/ER signaling in mammals^46–49^. By chromatin immunoprecipitation (ChIP) coupled with qPCR analysis in HepG2, we showed that PNPLA3-ERE1 was targeted for binding by ER-α after exposure to tamoxifen for 24 h (Fig. 4c), whereas PNPLA3-ERE2/ERE3 was not.

To examine whether PNPLA3-ERE1 functions as an enhancer for *PNPLA3*, we cloned the DNA region containing PNPLA3-ERE1 into the minimal promoter of a luciferase plasmid. Considering that estrogens can regulate target gene expression by binding to ERE or tethering with other transcription factors, we also included the adjacent activator protein 1 (AP-1)-binding site, and generated luciferase reporter plasmids containing ERE1- and AP-1-binding sites. We found that ERE1 is sufficient to increase the induction of luciferase in cells incubated with tamoxifen, but in the presence of the AP-1-binding site the induction is more robust (28%, *P* = not significant (NS) versus 58%, *P* < 0.01; Fig. 4d). These data suggest that PNPLA3-ERE1 is a functional element that enhances transcription when bound to ER-α in the presence of receptor agonists.

**Extended Data Fig. 7:**
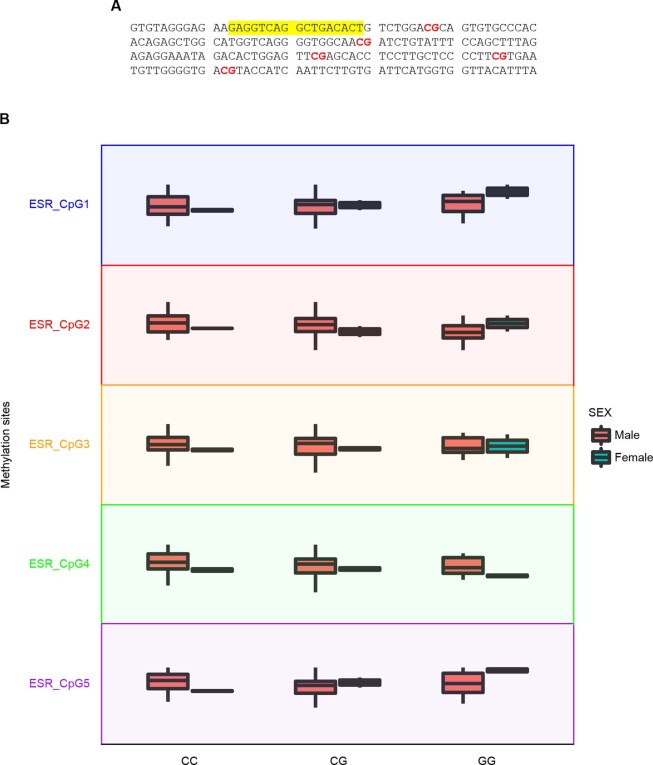
PNPLA3-ERE1 cfDNA methylation. Impact of sex and carriage of *PNPLA3* p.I148M variant on cfDNA methylation status of five different CpGs across the PNPLA3-ERE1 region in 90 participants of the Liver-Bible-2022 cohort. A) Schematic representation of CpG sites located immediately downstream of ERE1 (CpG1 at +24 bp, CpG2 at +66 bp, CpG3 at +110 bp, CpG4 at + 132 bp and CpG5 at + 149 bp). The ERE1 are underlined and CpGs in red. The region is chromosome 22: 44315438-44315637 (GRCh37/hg19 genome assembly). B) Boxplots showing CpG methylation ratio for each CpG in man (n = 85) and women (n = 6) stratified for *PNPLA3* p.I148M genotype. In the box and whisker plots, the line in the middle of the box represents the medians, tops and bottoms of the boxes represent the 25^th^ and 75^th^ quartiles respectively, and the whiskers showed the 5^th^ and 95^th^ percentile. The impact of the *PNPLA3* p.I148M variant on methylation was determined by generalized linear model (GLM), adjusted for sex. p=not significant for all comparisons. The impact of the variant was estimated by unadjusted generalized linear regression models, under an additive genetic model for the *PNPLA3* variant.

As the PNPLA3-ERE1 region contains five CpG sites (Extended Data Fig. 7a), we surveyed whether this element is regulated by epigenetic mechanisms by measuring its methylation in circulating cell-free DNA (cfDNA), as a fingerprint of epigenetic regulation of gene expression in 91 individuals in the Liver-Bible-2022 cohort (age 53.4 ± 6.7 years, 7% females, *PNPLA3* p.I148M genotype: 46% I/I, homozygous for the wild-type variant; 44% I/M, heterozygous; 10% M/M, homozygous for the risk variant). We found that PNPLA3-ERE1 was differentially methylated in women carrying the *PNPLA3* p.I148M variant, with one site being hypomethylated (CpG4) and three hypermethylated (Extended Data Fig. 7b). At multivariable analysis adjusted for age and CpG1 methylation status, the p.I148M variant and body mass index (BMI) were specifically associated with demethylation of CpG4 in women (*P* = 3 × 10^−8^ and *P* = 1 × 10^−8^, respectively), but not in men (*P* = 0.09 and *P* = 0.33, respectively). These data are consistent with hepatic gene expression data showing that *PNPLA3* is differentially upregulated in women carrying the p.I148M variant and that PNPLA3-ERE1 is involved in *PNPLA3* induction in response to ER activation.

### Role of PNPLA3-ERE1 in estrogen-dependent accumulation of PNPLA3 and lipids in hepatocytes

**Fig. 5:**
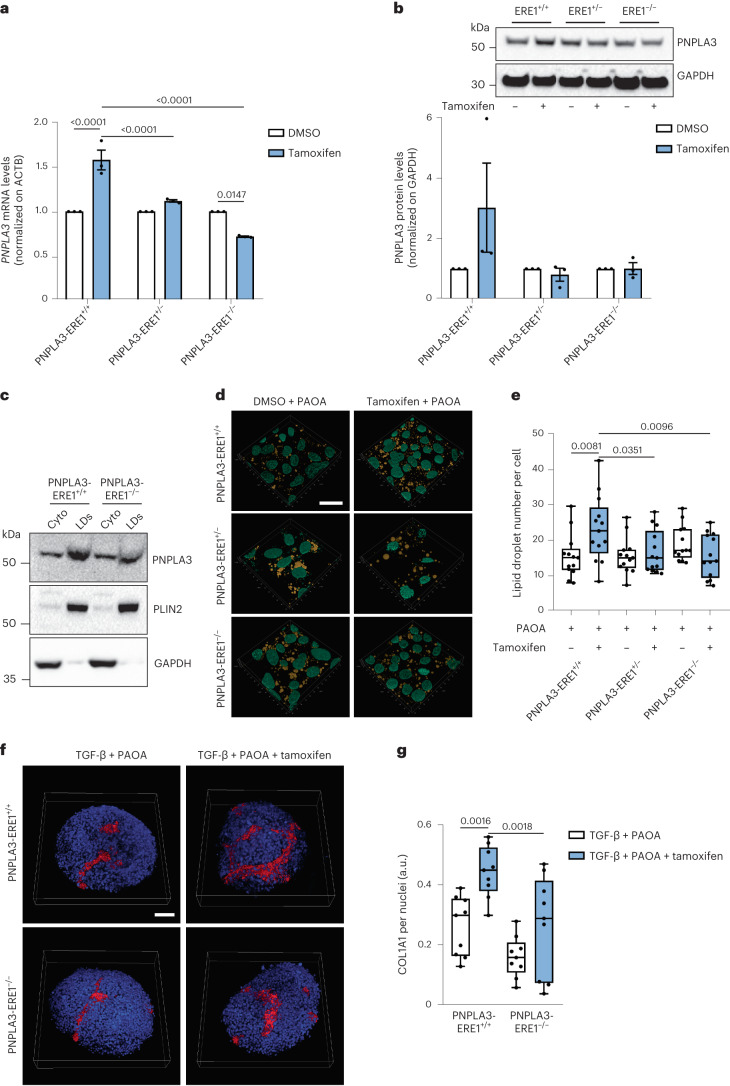
PNPLA-ER1 deletion hampers ER-α-mediated lipid droplet accumulation in HepG2 hepatocytes. **a**, RT-qPCR analysis of *PNPLA3* mRNA levels in HepG2 PNPLA3-ERE1^+/+^, PNPLA3-ERE1^+/−^ and PNPLA3-ERE1^−/−^ cells treated for 48 h with tamoxifen (10 μM) or DMSO as a negative control. **b**, Western blot analysis of PNPLA3 protein levels in HepG2 PNPLA3-ERE1^+/+^, PNPLA3-ERE1^+/−^ and PNPLA3-ERE1^−/−^ cells treated for 48 h with tamoxifen (10 μM) or DMSO as a negative control. (GAPDH used as a loading control was run on a different gel.) **c**, Western blot analysis of PNPLA3, PLIN2 and GAPDH proteins in cytosolic and lipid droplet fractions obtained from cells treated for 48 h with fatty acids (palmitic and oleic acids, both at 250 μM) and tamoxifen or DMSO as a negative control. **d**, Immunofluorescence staining with DAPI (cyan) and lipid droplets stained with Nile Red (yellow) of HepG2, PNPLA3-ERE1^+/−^ and PNPLA3-ERE1^−/−^ cells treated for 48 h with a mix of palmitic and oleic acids (250 μM each) and tamoxifen (10 μM) or DMSO as a negative control. Scale bar, 20 μm. **e**, Quantification of lipid droplet number by ImageJ normalized to nuclei number. Whiskers show minimum to maximum values, box bounds the 25th to 75th percentiles and the center the median value. **f**, Immunofluorescence staining of DAPI (blue) and COL1A1 (red) of 3D spheroids (HepG2/LX-2, 24:1) treated for 48 h with a mix of palmitic and oleic acids (250 μM each), TGF-β (10 ng ml^−l^) and tamoxifen (10 μM) or DMSO as a negative control. Scale bar, 50 μm. **g**, Quantification of COL1A1 levels by ImageJ normalized to DAPI quantification. Whiskers show minimum to maximum values, box bounds the 25th to 75th percentiles and the center the median value. Data in **a** and **b** are mean ± s.e.m. (*n* = 3 independent experiments). In **e** the data are mean ± s.e.m. (*n* = 12 images from 3 independent experiments). In **g** the data are mean ± s.e.m. (*n* = 9 images from 3 independent experiments). A two-way ANOVA was used followed by Bonferroni’s post hoc test. Source data

**Extended Data Fig. 8:**
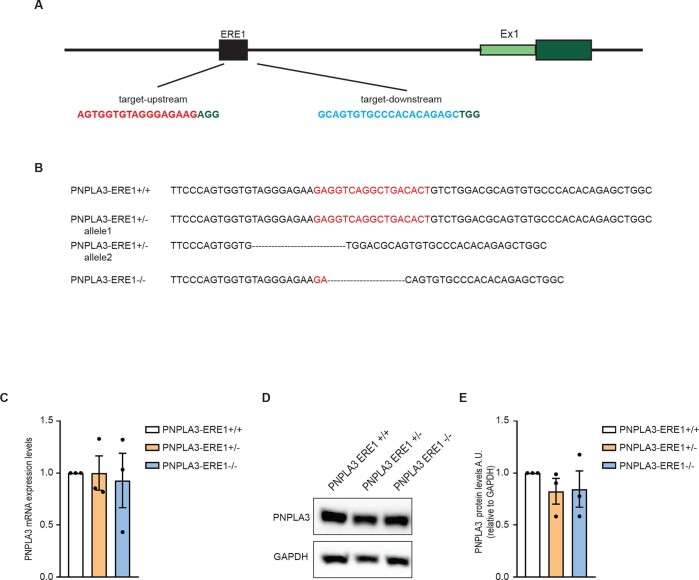
Generation of PNPLA3-ERE1 knock-out HepG2 hepatocytes. a) Schematic representation of *PNPLA3* gene promoter and sgRNA used to delete PNPLA3-ERE1 elements. b) Schematic representation of PNPLA3-ERE1 sequence and sanger sequencing results confirming the deletion of ER1 in heterozygosity and homozygosity. c) PNPLA3 expression level in PNPLA3-ERE1^+/+^, PNPLA3-ERE1^+/−^ and PNPLA3-ERE1^−/−^ HepG2 hepatocytes. d) PNPLA3 Western blot and e) relative quantification in PNPLA3-ERE1^+/+^, PNPLA3-ERE1^+/−^ and PNPLA3-ERE1^−/−^ HepG2 hepatocytes. Data in panel C and D are presented as mean ± SEM (n = 3 independent experiments).

To examine whether PNPLA3-ERE1 mediates in ER-α-dependent upregulation of PNPLA3, we engineered HepG2–Cas9^+^ cells^32^ to generate three syngeneic, independent, wild-type clones lacking PNPLA3-ERE1 in heterozygosity and homozygosity (Extended Data Fig. 8a,b: PNPLA3-ERE1^+/+^, PNPLA3-ERE1^+/−^ and PNPLA3-ERE1^−/−^, respectively). In PNPLA3-ERE1^+/+^ cells in response to the ER-α agonist PNPLA3 was induced by ~1.5-fold at mRNA and ~3-fold at protein levels (Fig. 5a,b). Despite the unaltered basal transcript abundance and similar protein levels (Extended Data Fig. 8c,d), after exposure to the ER-α agonist tamoxifen *PNPLA3* mRNA and protein expression failed to be upregulated in PNPLA3-ERE1^+/−^ hepatocytes, whereas they paradoxically tended to decrease in PNPLA3-ERE1^−/−^ cells exposed to tamoxifen compared with the corresponding untreated cells (Fig. 5a,b).

In addition, PNPLA3-ERE1^−/−^ cells treated with tamoxifen for 48 h accumulated lower levels of the PNPLA3 p.I148M protein in the lipid droplet subcellular fraction compared with PNPLA3-ERE1^+/+^ (Fig. 5c). In keeping with the notion that the p.I148M protein variant lacking enzymatic activity promotes intracellular fat retention, PNPLA3-ERE1^+/−^ and even more so PNPLA3-ERE1^−/−^ hepatocytes accumulated a lower number of intracellular lipid droplets compared with PNPLA3-ERE1^+/+^ hepatocytes in response to fatty acids and tamoxifen (Fig. 5d,e).

Finally, HepG2 PNPLA3-ERE1^+/+^ and PNPLA3-ERE1^−/−^ were cocultured with LX-2 cells (homozygous for the p.I148M variant) to form 3D spheroids. After treatment for 48 h with TGF-β and fatty acids (palmitic and oleic acids) to mimic fibrosing FLD, PNPLA3-ERE1^−/−^ spheroids showed a marked reduction in the upregulation of the synthesis and deposition of COL1A1 in response to tamoxifen compared with PNPLA3-ERE1^+/+^ spheroids (Fig. 5f,g).

All in all, these data suggest that estrogen-driven ER-α binding to PNPLA-ER1 can promote the synthesis and accumulation of *PNPLA3* p.I148M in intracellular lipid droplets in hepatocytes, facilitating the accumulation of lipids and triggering lipotoxicity and fibrogenesis.

## Discussion

In the present study, we demonstrated an interaction between sex and a common genetic variant, namely *PNPLA3* p.I148M, in the determination of the development and severity of FLD. This interaction was present at a genetically epidemiological and a molecular level and might help to explain the reasons why premenopausal women are protected against FLD, whereas, in a subset of women, a rapidly progressive disease may ensue at the menopause.

We started by showing that the *PNPLA3* p.I148M variant confers a larger increase in FLD risk in menopausal women (≥55 years), who had higher E_2_ levels than men, than in men in both at-risk individuals and the population-based UKBB cohort. This observation was specific for the *PNPLA3* p.I148M and not detected for the *TM6SF2* p.E167K or other main gene variants contributing to FLD susceptibility. Consistently, recent data on the prospective evaluation of the impact of the p.I148M variant on liver-related events in patients with histological FLD showed that the variant had a larger impact in women than in men^50^. Furthermore, a pilot experiment performed in mice knock-in for the *PNPLA3* p.I148M variant showed that females accumulated more triglycerides in the liver compared with males when fed with a high-sucrose diet for 8 weeks^30^. This finding has several implications. First, it identifies a driver of liver disease in women of menopausal age (≥55 years), women with metabolic dysfunction^11^. Silencing of hepatic *PNPLA3* mRNA encoding for p.I148M is now used in clinical trials as a therapeutic strategy against fibrotic NASH^21^. Therefore, the present results imply that hepatic PNPLA3 downregulation may be even more effective in reducing hepatic fat accumulation and liver damage in women than in men. Second, our study highlights how menopausal women (≥55 years), so far neglected in clinical studies of liver disease, when carrying the *PNPLA3* p.I148M variant may become a clinically distinct and relevant subset to target with a precision therapy approach^15^. Third, from a genetic epidemiology perspective, our finding provides a robust demonstration of a strong interaction, with a multiplicative effect, between carriage of a disease risk variant with a large effect size and female sex in determining a common disease. The interaction between the *PNPLA3* p.I148M variant and female sex provides a proof of principle that epistasis contributes to the genetic architecture of common traits such as FLD. Last, but not least, we reveal the molecular mechanism underlying the genetic epidemiological interaction that encompasses a direct *PNPLA3* upregulation by estrogen signaling. This mechanism may account for previous epidemiological observations and sex-specific associations between the presence of the *PNPLA3* p.I148M variant on hepatic phenotypes observed in mice^29^, and may be useful to improve the design of future experimental studies, for example, by taking into consideration variations in hormonal levels in rodent models. Furthermore, in patients with advanced chronic liver disease, there is evidence for an increase in the estrogen to androgen ratio^51^ due to the impaired hepatic metabolism of steroid hormones. As ER-α activity can be pharmacologically modulated and ER-α is also transcriptionally active in men^8^, additional studies are required to evaluate whether these findings may account for the acceleration of disease progression at a late stage in some men with FLD.

The presence of a nonenzymatically active *PNPLA3* p.I148M on lipid droplets is believed to represent the fundamental mechanisms by which the genetic variant causes liver lipid accumulation^21^. Starting from the observation that PNPLA3 is more expressed in the liver of women than of men, in particular in those with higher E_2_ levels, and that EREs are located at the distal promoter of the *PNPLA3* gene, we hypothesized that the mechanism leading to increased effect of the variant in women was the result of enhanced protein expression mediated by ERs. Consistent with this hypothesis, we showed that ER-α agonists bound to PNPLA3-ERE1 lead to higher transcription and synthesis of PNPLA3. Consequently, in human HepG2 hepatoma cells, homozygotes for the p.I148M variant, PNPLA3 upregulation by ER-α agonists was associated with intracellular accumulation of neutral lipids and an increase in lipid droplet number. The upregulation of PNPLA3 by E_2_ was confirmed in HLOs and HepaRG cells. However, in HepaRG cells that bear two enzymatically active, wild-type *PNPLA3* alleles, the upregulation did not result in intracellular lipid accumulation. Genetic deletion of PNPLA3-ERE1 in HepG2 cells completely abrogated ER-α-mediated PNPLA3 upregulation, intracellular lipid accumulation and fibrogenesis, indicating that this ERE is required for ER-α-dependent PNPLA3 induction. At the same time, PNPLA3-ERE1 exposed the beneficial impact of estrogen signaling on intracellular lipid metabolism occurring in the absence of accumulation of the PNPLA3 p.I148M variant protein. Indeed, in premenopausal women E_2_ reduces the synthesis and increases the oxidation of fatty acids in the liver^32,33^, resulting in protection against FLD. This process is mediated by hepatic ER-α, the most abundant ER in hepatocytes^40,52^.

The larger phenotypic expression of the p.I148M variant in menopausal versus premenopausal women remains an apparent conundrum, because it does not fit with the relatively lower E_2_ levels after menopause. However, FLD is a multifactorial disease due to several drivers, including factors other than genetic predisposition and sex hormones: epigenetic modifiers, diet, lifestyle and microbiota composition. With respect to the sexual dimorphism, young premenopausal women show a lower incidence of FLD, consistent with a pivotal role exerted by estrogens at least partly through hepatic ER-α in counteracting FLD development and progression^52–54^. The relatively lower E_2_ levels in menopausal compared with premenopausal women negatively affect the regulation of hepatic metabolism, favoring de novo lipogenesis, lipid import and deposition in the liver, and inhibiting lipid catabolism and export from the liver, while still resulting in higher induction of PNPLA3 compared with men. In addition, the relative reduction of circulating E_2_ in postmenopausal women negatively affects the regulation of systemic metabolism (that is, decreased insulin sensitivity; changes in adiposity and adipose tissue distribution, and increased flux of fatty acid from adipose tissue to the liver as a consequence of the lower inhibition of adipose tissue lipolysis), further contributing to increased lipid accumulation in the liver and exposing the effect of the *PNPLA3* p.I148M on FLD development. In keeping with this hypothesis, we showed that the different levels of circulating E_2_ observed in pre- and postmenopausal women and men were mirrored by the hepatic *PNPLA3* mRNA levels, which tended to remain higher in menopausal women compared with men.

Together, in women after the menopause the decrease in estrogen levels predisposes to FLD onset together with other metabolic alterations, including increased adiposity and insulin resistance. In carriers of the p.I148M variant, the persistence of relatively high E_2_ resulting from estrone conversion in hepatocytes can synergize with inflammation-mediated mechanisms^47,52,53^ and insulin resistance acting via SREBP1c, resulting in induction and accumulation of the p.I148M variant protein and consequently in hepatic fat accumulation. Indeed, adiposity and insulin resistance are the main triggers of the phenotypic expression of the *PNPLA3* p.I148M variant^6^. Furthermore, increased levels of circulating amino acids can increase ER-α activity independently of E_2_ levels during insulin resistance^54^. However, we cannot rule out that other factors contribute independently of ER-α to the more severe hepatic expression of the *PNPLA3* p.I148M variant in women than in men.

A limitation of the present study is that these findings may be not applicable to non-European ancestry, because all analyses were conducted on cohorts mainly composed of European individuals, and these results should be confirmed in larger cohorts of patients at high risk of liver events, adopting a prospective approach. In addition, the specific hormones with agonist/antagonist activity on the ER-α–PNPLA3-ERE1 axis in premenopausal versus postmenopausal women, in men and during liver disease progression need further investigations in in vivo models testing the impact of sex and estrogens on the phenotypic expression of the *PNPLA3* p.I148M variant. Finally, it remains to be demonstrated whether the larger phenotypic expression of the *PNPLA3* p.I148M variant in menopausal versus premenopausal women, despite the lower E_2_ levels, is exclusively related to the worsening of insulin sensitivity in older women, or that other factors may be involved.

In conclusion, we observed a multiplicative interaction between the *PNPLA3* p.I148M variant and female sex in determining FLD risk that is particularly evident in postmenopausal women. We showed that the underlying mechanism to this interaction probably encompasses an estrogen-mediated upregulation of PNPLA3 via ER-α and a specific ERE at an enhancer site in the *PNPLA3* promoter. We pinpointed that this PNPLA3-ERE1 DNA region is required to allow accumulation of PNPLA3 on lipid droplets, leading to the expansion of their number in response to ER-α agonists. These findings highlight a specific mechanism contributing to the sexual dimorphism of the most common cause of liver disease in the population and may be used to design precision medicine approaches targeted to women.

## Methods

### Clinical cohorts

The cross-sectional Liver Biopsy Cohort has previously been described^22,55^. Briefly, up to 1 July 2018, a total of 1,861 adult individuals of European descent were consecutively enrolled from Italian and Finnish referral centers. Inclusion criteria were liver biopsy for suspected NASH or severe obesity, availability of clinical data and consent. Individuals with at-risk alcohol intake (≥30/20 g per d in men/women) or other causes of liver disease (including viral or autoimmune hepatitis, drug-induced liver injury, hemochromatosis, α_1_-antitrypsin deficiency and other monogenic liver diseases) were excluded.

The transcriptomic cohort consisted of a subgroup of 125 severely obese individuals from the Liver Biopsy Cohort, who underwent a percutaneous liver biopsy performed during bariatric surgery at the Milan center for clinical staging of liver disease severity and for whom sufficient material for extraction of high-quality RNA was available^56^. Individuals with at-risk alcohol intake (>30/20 g per d in men/women), viral and autoimmune hepatitis or other causes of liver disease were excluded.

The severe metabolic FLD case–control cohort (*n* = 4,374), included 511 patients with advanced fibrosis (stage F3–F4) and/or HCC due to nonalcoholic fatty liver disease (NAFLD) associated with metabolic dysfunction without at-risk alcohol intake and population-matched controls with available genotyping due to the inclusion in the EPIDEMIC and SERENA cohort studies and 3,863 population controls (FOGS study)^32,57^. Advanced liver fibrosis was diagnosed in the presence of pathognomonic clinical, biochemical or imaging evidence of cirrhosis and/or histological evidence of advanced liver fibrosis and/or fibrosis-4 index for liver fibrosis (FIB-4) score ≥1.3 plus liver stiffness measurement (LSM) by fibroscan ≥8 kPa^58^. HCC was diagnosed according to current clinical practice guidelines^59^. Exclusion criteria were the same as above.

The Liver-Bible-2022 cohort included 1,142 individuals with metabolic dysfunction^60–62^, who were consecutively enrolled from July 2019 to July 2022, and for whom information on genomic data was available. These were apparently healthy blood donors, aged 40–65 years, who were selected for a comprehensive liver disease, metabolic and cardiovascular screening, owing to the presence of at least three metabolic risk abnormalities, from overweight/obesity (defined as BMI ≥ 25 kg m^−2^), hypertension (blood pressure ≥130/85 mmHg or antihypertensive treatment), dysglycemia (fasting glucose level ≥100 mg dl^−1^ or use of glucose-lowering agents), low plasma high-density lipoprotein (HDL)-cholesterol (<45 mg dl^−1^ in men and <55 mg dl^−1^ in women) or high plasma triglycerides (≥150 mg dl^−1^ or lipid-lowering treatment). Individuals with chronic degenerative diseases (such as advanced kidney disease, cirrhosis or active cancer), except for well-controlled arterial hypertension, treated hypothyroidism and well-compensated type 2 diabetes (T2D) not requiring pharmacotherapy (except for metformin), were excluded from the cohort at first evaluation. The overall goal of this ongoing biobank study was primarily to examine the role of genetic factors and other noninvasive biomarkers of NAFLD in the risk prediction of cardiometabolic diseases, to provide the framework to design precision medicine approaches to prevent these cardiometabolic conditions.

The menopause status in the study cohorts was defined based on clinical history and evaluation, and women missing menopause status aged >55 years were classified as menopausal. All women participating in the Liver-Bible-2022 were recalled to re-evaluate menopausal status and hormonal therapy.

HLOs were isolated from surgical samples obtained from patients who underwent liver transplantation (from the explanted liver) or liver resection for malignant (HCC, biliary tract carcinoma and metastatic colon cancer) and nonmalignant liver disorders (hepatic adenomas, angiomas and biliary disorders) at the Fondazione IRCCS Ca’ Granda of Milan (approved under the REASON study protocol). Patients had to be negative for active chronic viral infections (hepatitis B and C virus, and human immunodeficiency virus), and for the present study had to be negative for monogenic liver disorders.

Informed written consent was obtained from each patient and the study protocol was approved by the Ethical Committee of the Fondazione IRCSS Ca’ Granda (EPIDEMIC-TERT no. 1882_2013; Perspective-SERENA no. 485_2017; REASON no. 728_2021; Liver-Bible no. 896_2022) and the other involved Institutions and conformed to the ethical guidelines of the 1975 Declaration of Helsinki. The clinical features of individuals included in the cross-sectional Liver Biopsy Cohort, transcriptomic, severe metabolic FLD case–control and Liver-Bible-2022 cohorts stratified by sex are presented in Table 1.

**Table 1:** Clinical features of individuals included in the study cohorts stratified by sex

	Liver biopsy cohort (*n* = 1,861)		
	Women (*n* = 920, 49.4%)	Men (*n* = 941, 50.6%)	*P* value
Age (years)	45.1 ± 14.0	42.9 ± 16.0	0.002
Menopause (no/transition/yes)^a^	379/273/268 (41.2/29.7/29.1)	N/A	N/A
BMI (kg m^−2^)	38.1 ± 8.7	31.6 ± 8.0	<0.0001
Severe obesity	406 (43.8)	156 (16.6)	<0.0001
T2D/IFG (yes)	225 (24.5)	258 (27.7)	0.16
Total cholesterol (mg dl^−1^)	192 ± 46	190 ± 42	0.89
Triglycerides (mg dl^−1^)	130 ± 64	146 ± 84	<0.0001
HDL-cholesterol (mg dl^−1^)	52 ± 16	44 ± 11	<0.0001
ALT (IU l^−1^)	54 (34-80)	30 (19-48)	<0.0001
AST (IU l^−1^)	34 (24-47)	22 (17-33)	<0.0001
NASH (yes)	249 (27.1)	370 (39.4)	<0.0001
Clinically significant fibrosis (yes)	161 (17.5)	259 (27.6)	<0.0001
*PNPLA3*, p.148M/M	128 (13.9)	142 (15.1)	0.51

	Transcriptomic cohort (*n* = 125)		
	Women (*n* = 107, 85.6%)	Men (*n* = 18, 14.4%)	*P* value
Age (years)	43.9 ± 10.5	42.5 ± 10.7	0.61
Menopause (no/transition/yes)^b^	55/35/17 (51.4/32.7/15.9)	N/A	N/A
BMI (kg m^−2^)	40.4 ± 7.4	42.3 ± 6.4	0.29
T2D/IFG (yes)	13 (12.2)	2 (11.1)	0.87
Total cholesterol (mg dl^−1^)	208 ± 47	214 ± 43	0.71
Triglycerides (mg dl^−1^)	132 ± 59	132 ± 59	0.48
HDL-cholesterol (mg dl^−1^)	56 ± 14	46 ± 9	0.031
ALT (IU l^−1^)	19 (14-26)	39 (24-51)	0.025
AST (IU l^−1^)	17 (15-20)	26 (17-31)	0.37
NASH (yes)	8 (7.5)	6 (33.3)	0.001
Clinically significant fibrosis (yes)	4 (3.7)	3 (16.7)	0.20
*PNPLA3*, p.148M/M	8 (7.5)	1 (5.6)	0.08

	Severe FLD case–control cohort (*n* = 4,374)		
	Women (*n* = 1,383, 31.6%)	Men (*n* = 2,991, 68.4%)	*P* value
Age (years)	44.2 ± 15.4	47.3 ± 13.8	<0.0001
Menopause (no/transition/yes)^c^	1,089/897/1,006 (36.4/30.0/33.6)	NA	NA
Advanced fibrosis (yes)	168 (12.1)	343 (11.5)	0.51
HCC (yes)	38 (2.8)	133 (4.4)	0.007
*PNPLA3*, p.148M/M	171 (12.4)	1303 (10.1)	0.028

	Liver-Bible-2022 cohort (*n* = 1142)		
	Women (*n* = 191, 16.7%)	Men (*n* = 951, 83.3%)	*P* value
Age (years)	53.9 ± 5.9	53.9 ± 6.5	0.98
Menopause (no/transition/yes)^d^	69/10/112 (36.1/5.2/58.7)	N/A	N/A
BMI (kg m^−2^)	28.8 ± 3.7	28.5 ± 3.0	0.26
T2D/IFG (yes)	26 (13.6)	56 (5.9)	0.0006
Total cholesterol (mg dl^−1^)	208 ± 34	202 ± 33	0.034
Triglycerides (g dl^−1^)	147 ± 62	166 ± 86	0.0006
HDL-cholesterol (mg dl^−1^)	52 ± 10	44 ± 9	<0.0001
ALT (IU l^−1^)	21 (16–27)	28 (22–37)	<0.0001
AST (IU l^−1^)	20 (17–23)	23 (20–27)	<0.0001
NAFLD (controlled attenuation parameter ≥ 275 dB m^−1^)	80 (41.9)	469 (49.5)	0.057
Clinically significant fibrosis (LSM ≥ 8 kPa)	1 (0.5)	25 (2.6)	0.11
*PNPLA3*, p.148M/M	17 (8.9)	75 (7.9)	0.31

	cfDNA from patients of Liver-Bible-2022 cohort (*n* = 91)		
	Women (*n* = 6, 6.6%)	Men (*n* = 85, 93.4%)	*P* value
Age (years)	53.8 ± 6.8	53.4 ± 6.8	0.91
Menopause (yes)	4 (66.7)	N/A	N/A

	UK Biobank cohort (*n* = 347,127)		
	Women (*n* = 186,625, 53.8%)	Men (*n* = 160,502, 42.2%)	*P* value
Age (years)	56.6 ± 7.9	57.0 ± 8.1	<0.0001
Menopause (yes)	134,358 (72.0)	N/A	N/A
E_2_ (pmol l^−1^)	538 ± 476	228 ± 79	<0.0001
BMI (kg m^−2^)	27.0 ± 5.1	27.8 ± 4.2	<0.0001
T2D (yes)	12,567 (6.7)	19,300 (12.0)	<0.0001
Total cholesterol (mg dl^−1^)	228 ± 43	213 ± 43	<0.0001
Triglycerides (g dl−^1^)	137 ± 75	175 ± 100	<0.0001
HDL-cholesterol (mg dl^−1^)	62 ± 15	50 ± 11	<0.0001
ALT (IU l^−1^)	18 (9-27)	20 (9-32)	<0.0001
AST (IU l^−1^)	23 (16-30)	24 (16-32)	<0.0001
NAFLD, hepatic fat content ≥5.5% (*n* = 6,318)	2,416 (14.0)	3,902 (23.7)	<0.0001
*PNPLA3*, p.148M/M	7,571 (4.7)	8,814 (4.7)	0.93

Upper panel: 1,861 individuals at risk of NAFLD in the cross-sectional Liver Biopsy Cohort. Lower panel: 125 dysmetabolic individuals used as transcriptomic cohort. The impact of the sex was estimated by unadjusted logistic regression models. AST, aspartate transaminase; N/A, not available.

^a^No woman was on estrogen therapy and three had polycystic ovary syndrome.

^b^One woman was on estroprogestinic therapy and one had polycystic ovary syndrome.

^c^No woman among cases was on hormonal therapy.

^d^Seven women (5.7%) among cases at menopause or postmenopause were on hormonal therapy.

### General population cohort

To investigate the impact of the interaction between the genetic background and female sex (participants self-reported their biological sex at the time of enrollment, which had to be consistent with genetically determined sex for inclusion in this analysis) at the population level, we consulted the UK Biobank (UKBB) cohort database (application approval no. 37142). The UKBB includes European individuals, where hard endpoints are defined by using a diagnosis code and genetic data are available for *n* ≈ 450,000 individuals, aged between 40 years and 69 years, who visited 22 recruitment centers throughout the UK between 2006 and 2014. The UKBB study received ethical approval from the National Research Ethics Service Committee of North West Multi-Centre Haydock (reference no. 16/NW/0274)^63,64^. For the present study, we restricted our analyses to unrelated European individuals (*n* = 365,449) from the UKBB. The menopausal status was defined based on datafield 2,724. Furthermore, those women with missing menopausal status who underwent bilateral oophorectomy (datafield 2,834) or were >55 years were also classified as postmenopausal^65^. The clinical features of individuals included in the UKBB cohort stratified by sex are presented in Table 1.

### Transcriptomic and bioinformatics analysis

Transcriptomic and bioinformatic analyses were performed as previously described^56^. Briefly, RNA was extracted using the RNeasy mini-kit (QIAGEN) and integrity assessed through Agilent 2100 Bioanalyzer. RNA sequencing (RNA-seq) was exploited in paired-end mode with a read length of 150 nt using the Illumina HiSeq 4000 (Novogene). Raw reads were aligned on the GRCh37 reference genome using STAR mapper. Reads count, according to ENSEMBL human transcript reference assembly v.75, was performed using the RSEM package^66^. Count normalization and differential gene expression analysis were performed using the DESeq2 package^67^.

To examine the determinant of *PNPLA3* expression and the interaction between female sex and the *PNPLA3* variant on hepatic gene expression, we performed class comparison for the presence of the p.I148M variant in the 107 women included in transcriptomic cohort (56 carriers; analysis was adjusted for age and RNA extraction batch). From this analysis, we identified 1,619 differentially expressed genes (DEGs) at a genome-wide level. As a second step, we analyzed the DEGs for interaction effect in the whole cohort (female sex × p.I148M variant carriage, adjusted for age and batch).

To identify differentially expressed pathways, we used *P* ≤ 0.05 as a cutoff for DEG inclusion criteria for IPA (QIAGEN: www.qiagen.com/ingenuity) and GSEA (http://www.broad.mit.edu/gsea). GSEA was performed in the preranked mode, with the dataset (v.7.4), in which phenotypes were permuted 1,000× to obtain stable analysis results.

### Animal model

In the present study, mice were fed freely with a standard diet (4RF21 standard diet, Mucedola) and provided with filtered water. The animal room was maintained within a temperature range of 22–25 °C and relative humidity 50 ± 10% and under an automatic cycle of 12-h light:12-h dark (lights on at 7:00 a.m.). To avoid any possible confounding effect owing to the circadian rhythm or feeding status, the mice were euthanized in the early afternoon after 6 h of fasting. Female mice were collected when in the proestrus (high estrogen levels) or metestrus (low estrogen levels) phase of the estrous cycle after vaginal smear analysis was done at 9:00 a.m.

All animal experimentation was done in accordance with the ARRIVE and European guidelines for animal care and use of experimental animals. The animal study protocol was approved by ‘Istituto Superiore di Sanità-Ministero della Salute Italiano’ (protocol code 1272/2015-PR, date of approval 15 December 2015).

### Cellular models

HepG2 (American Type Culture Collection (ATCC), catalog no. HB-8065), LX-2 (Sigma-Aldrich, catalog no. SCC064) and 293T (ATCC, catalog no. CRL-3216) cells were cultured at 37 °C and 5% CO_2_ in Dulbecco’s modified Eagle’s medium (DMEM) high glucose (Thermo Fisher Scientific, catalog no. 11965084) supplemented with 10% fetal bovine serum (FBS; Thermo Fisher Scientific, catalog no. 10270106), 1% glutamine (Thermo Fisher Scientific, catalog no. 25030081) and 1% penicillin–streptomycin (Thermo Fisher Scientific, catalog no. 15140122). HepaRG (Thermo Fisher Scientific, catalog no. HPRGC10) was grown at 37 °C and 5% CO_2_ in Williams’ medium supplemented with 10% FBS, 1× ITS-X (Thermo Fisher Scientific, catalog no. 51500056), 50 μM hydrocortisone hemisuccinate (Sigma-Aldrich, catalog no. H2270) and 1% penicillin–streptomycin.

All cell lines were routinely tested to exclude *Mycoplasma* contamination using MycoAlert PLUS *Mycoplasma* Detection Kit (Lonza, catalog no. LT07-710).

Cells were treated with the following reagents in the described experimental procedures: 17β-estradiol (E_2_; Sigma-Aldrich, catalog no. E8875), tamoxifen (Sigma-Aldrich, catalog no. T5648), PPT (Sigma-Aldrich, catalog no. H6036), DPN (Sigma-Aldrich, catalog no. H5915) and G-1 (Bio-Techne, catalog no. 3577). To induce fat overloading of cells, HepG2 cell clones were seeded at a density of 50,000 cells cm^−2^ and after 48 h cells were starved for another 24 h, whereas HepaRG cell clones were starved for 24 h when they enriched the confluence. Then cells were exposed to a mixture of long-chain fatty acids (FAs: oleate and palmitate) at a 2∶1 ratio conjugated to bovine serum albumin (BSA; Sigma-Aldrich, catalog no. 126575). Stock solutions of 5 mM oleic acid (Sigma-Aldrich, catalog no. O1383) and 5 mM palmitic acid (Sigma-Aldrich, catalog no. P5585) were prepared by mixing FAs in a medium containing 10% BSA overnight at 40 °C.

HLOs were obtained and differentiated following a previously published protocol^44^. Human liver samples were kept before processing at 4 °C in basal medium: advanced DMEM/F-12 (Thermo Fisher Scientific, catalog no. 12634010) supplemented with 1% penicillin–streptomycin (Thermo Fisher Scientific, catalog no. 15070063), 1% Glutamax (Thermo Fisher Scientific, catalog no. 35050061) and 10 mM Hepes (Thermo Fisher Scientific, catalog no. 15630056). Samples were manually minced with surgical knives and washed twice with 10 ml of wash medium (DMEM high glucose supplemented with 1% FBS and 1% penicillin–streptomycin). Tissue was then further dissociated by enzymatic digestion with 0.125 mg ml^−1^ of collagenase (Thermo Fisher Scientific, catalog no. 17104019) and 0.125 mg ml^−1^ of dispase II (Thermo Fisher Scientific, catalog no. 17105041) and 0.1 mg ml^−1^ of DNase I (Sigma-Aldrich, catalog no. D2821) at 37 °C for no more than 90 min to obtain an 80–100% single-cell solution. The solution was then filtered through a 70-µm-pore cell strainer and the volume was increased to 50 ml with ice-cold wash medium before centrifuging at 300*g* for 5 min at 8 °C. Pellets were resuspended in 15 ml of wash medium and washed twice with 15 ml of wash medium and once with 10 ml of basal medium (each time pelleting the material by centrifuging at 300*g* for 5 min at 8 °C). Cells were resuspended in Cultrex BME (Bio-Techne, catalog no. 3432-010-01) and seeded in 40 µl of Matrigel drops in 24-well, low-attachment plates. Drops were overlayed with 500 µl of isolation medium: basal medium supplemented with 1× N21-MAX supplement (Bio-Techne, catalog no. AR008), 1× N-2 supplement (Bio-Techne, catalog no. AR009), 1 mM *N*-acetylcysteine (Sigma-Aldrich, catalog no. A9165), 500 µg ml^−1^ of R-spondin1 (Peprotech, catalog no. 120-38), 10 mM nicotinamide (Sigma-Aldrich, catalog no. N0636), 10 nM recombinant human [Leu]-Gastrin I (Sigma-Aldrich, catalog no. G9145), 50 ng ml^−1^ of recombinant human epidermal growth factor (EGF; Peprotech, catalog no. AF-100-15), 100 ng ml^−1^ of recombinant human fibroblast growth factor 10 (FGF-10; Peprotech, catalog no. 100-26), 5 µM A83-01 (Bio-Techne, catalog no. 2939), 10 µM forskolin (Bio-Techne, catalog no. 1099), 25 ng ml^−1^ of recombinant human growth factor (HGF; Peprotech, catalog no. 100-39H), 25 ng ml^−1^ of Noggin (Peprotech, catalog no. 120-10 C), 50 ng ml^−1^ of Wnt3a (Peprotech, catalog no. 315-20) and 10 µM Y-27632 (Stemcell Technologies, catalog no. 72302). After 3–4 d, the isolation medium was replaced with an expansion medium (lacking Noggin, Wnt3a and Y-27632).

Then, 7 d before differentiation, expansion medium was supplemented with 25 ng ml^−1^ of bone morphogenetic protein 7 (BMP-7; Peprotech, catalog no. 120-03P), then organoids were kept for 11–13 d in differentiation medium: advanced DMEM/F-12 medium supplemented with 1% N21-MAX, 1% N-2, 50 ng ml^−1^ of EGF, 10 nM astrin-1, 25 ng ml^−1^ of HGF, 100 ng ml^−1^ of FGF-19 (Peprotech, catalog no. 100-32), 500 nM A83-01, 10 µM DAPT (Bio-Techne, catalog no. 2634), 25 ng ml^−1^ of BMP-7 and 30 µM dexamethasone (Bio-Techne, catalog no. 1126). Fresh differentiation medium was given every other day.

### PNPLA3-ER1 ERE genetic deletion and mutagenesis in HepG2 hepatocytes

To investigate the role of the ERE in overexpression of PNPLA3, we disrupted ERE-α using the clustered regularly interspaced short palindromic repeats (CRISPR)–Cas9 method. A stable HepG2–Cas9 cell line was generated as previously described^32^. Briefly, single-guide RNAs (sgRNAs) were designed using the online tool E-CRISP (http://www.e-crisp.org/E-CRISP) and cloned in the pGL3-U6-sgRNA-PGK-puromycin vector (Addgene, catalog no. 51133)^68^. HepG2 cells were genome edited by expression of the doxycycline-inducible Cas9 combined with the sgRNA construct and PNPLA3-ERE1^+/−^ and PNPLA3-ERE1^−/−^ clones were generated following a homologous directed repair approach. HepG2 cells were naturally homozygous for the *PNPLA3* p.I148M variant. Proper clones containing desired *PNPLA3* mutations were selected by applying a puromycin resistance gene as the selection marker (Thermo Fisher Scientific, catalog no. A1113802), cultured in the separated dishes and, after collecting genomic DNA, the surveyor assay using the T7 endonucleases (NEB, catalog no. E3321) was performed to confirm locus-specific efficiency of genome editing. Positive clones were sequenced by Sanger to confirm *PNPLA3* gene-promoter mutations. Oligonucleotides used in these experiments are listed in Supplementary Table 5.

### RNA isolation and RT-qPCR

Total RNA was isolated from the HepG2 cell line using TRIzol reagent (Thermo Fisher Scientific, catalog no. 15596026) following the manufacturer’s instruction. RNA concentration and purity were verified using a NanoDrop ND-100 spectrophotometer (NanoDrop Technologies). For the real-time quantitative PCR (RT-qPCR) assay, complementary DNA was synthesized from 1,000 ng of total RNA with SuperScript IV VILO (Thermo Fisher Scientific, catalog no. 11766050).

Gene expression levels were measured using Fast SYBR Green Master Mix (Thermo Fisher Scientific, catalog no. 4385610) on an ABI 7500 fast thermo cycler (Thermo Fisher Scientific). All reactions were performed in triplicate. The relative expression levels of the selected targets were normalized to *ACTB* mRNA levels. All primers used for RT-qPCR analysis are listed in Supplementary Table 5.

### Protein extraction and western blot analysis

Total protein extracts were obtained as follows: cells were washed twice with cold phosphate-buffered saline (PBS), harvested by scrapping in 1 ml of cold PBS and centrifuged for 5 min at 300*g*. Cell pellets were lysed by the addition of 5× (v/v) ice-cold radioimmunoprecipitation buffer for 20 min at 4 °C. Lysates were cleared by centrifugation for 10 min at 12,000*g* and 4 °C and the supernatant was collected on ice. The protein concentration of lysates was determined using Pierce BCA Protein Assay Kit (Thermo Fisher Scientific, catalog no. 23227), according to the manufacturer’s instructions. The absorbance was measured at *λ* = 595 nm using the Tecan spectrophotometer (SAFAS). Values were compared with a standard curve obtained from the BSA dilution series.

For each sample a mix was prepared containing 20 μg of protein, 1× LDS (lithium dodecylsulfate) sample buffer (Thermo Fisher Scientific, catalog no. NP0007) and 1× sample reducing agent (Thermo Fisher Scientific, catalog no. NP0009). For each sample a protein mix was prepared sufficient to run at least three independent gels. For western blot analysis, protein mixtures were boiled at 95 °C and loaded on to precast Bolt 4–12% Bis–Tris Plus gels (Thermo Fisher Scientific, catalog no. NW04122BOX) and run in Bolt MES running buffer (Thermo Fisher Scientific, catalog no. B0002). After electrophoresis, proteins were transferred to a nitrocellulose membrane. Membranes were blocked in PBS + Tween 20 (PBS-T) containing 5% milk (blocking buffer; Sigma-Aldrich, catalog no. 170-6404), for 1 h at room temperature (RT) and then incubated with the indicated primary antibody diluted in blocking buffer overnight at 4 °C with agitation. The membrane was then washed 3× with PBS-T, each time for 5 min, followed by incubation with secondary antibody horseradish peroxidase (HRP) conjugated for 1 h at RT. ECL reagents (GE Healthcare, catalog no. RPN2232) was used to initiate the chemiluminescence of HRP. The chemiluminescent signal was captured using the ChemiDoc system (BioRad). All antibodies used for western blot analysis are listed in Supplementary Table 6.

### Detection of EREs

The promoter region of *PNPLA3* gene was analyzed using the Dragon ERE Finder 6.0 (ref. ^69^) algorithm to identify the presence of putative ERE.

### ChIP

HepG2 cells were starved in phenol-free DMEM overnight and two groups of cells were prepared: treated cells and untreated cells with 5 µM tamoxifen with two different timepoints of 8 h and 24 h. ChIP assay was carried out using Pierce Agarose ChIP Kit following the manufacturer’s instructions (Thermo Fisher Scientific, catalog no. 26156). First, formaldehyde was added for the crosslinking process at a final concentration of 1% directly to the cells and the reaction was quenched by adding glycine 1×. The cell lysate was resuspended in the Mnase digestion buffer and micrococcal nuclease was added to share the DNA. ChIP assays were performed by overnight immunoprecipitation at 4 °C on a rocking platform with anti-RNA polymerase II antibody as a positive control (Thermo Fisher Scientific, catalog no. 1862243), normal rabbit immunoglobulin (Ig)G as a negative control (Thermo Fisher Scientific, catalog no. 1862244) and ER-α antibody as a target-specific IP (Cell Signaling, catalog no. 8644S). Protein A/G agarose was added to collect the histone–antibody complex. Input and immunoprecipitated chromatin were incubated at 65 °C for 40 min to reverse crosslinks. After proteinase K digestion, DNA was extracted using a spin column. Then, 1 μl of each sample was assayed by RT-qPCR. As a positive control, we analyzed a region of the hepcidin (HAMP) promoter known to be regulated by ER-α (from −2,559 to −2,002 before the TSS)^70^. DNA was analyzed by RT-qPCR using SYBR GreenER kit (Invitrogen). All experimental values were shown as a percentage of input. To take into account background signals, we subtracted the values obtained with one IgG alone to the relative ChIP signals.

All primers for detecting EREs in *PNPLA3* are listed in Supplementary Table 5.

### Construct generation, transfection and luciferase reporter assay

The region spanning ER-α-binding elements (−112 to +371 bp) were amplified by PCR from human gDNA by using Platinum SuperFi II Master mix (Thermo Fisher Scientific, catalog no. 12368010) according to the manufacturer’s instruction, and inserted into the promoter region of the pGL4 basic vector (Promega, catalog no. E6651) at the NheI-XhoI restriction site.

Transfection of plasmids was performed using Lipofectamine 3000 reagent (Thermo Fisher Scientific, catalog no. L3000001) according to the manufacturer’s instructions. The luciferase constructs and pRL-TK, which encodes *Renilla* luciferase, were cotransfected into HEK293 cells in a 24-well plate. After 48 h, the cells were lysed in a single freeze–thaw cycle in a passive lysis buffer and the luciferase activities in the supernatant were measured using Dual-Luciferase Reporter Assay System kits (Promega, catalog no. E1910). The relative activity of luciferase was determined using the *Renilla* luciferase signal as the reference.

### Intracellular lipid staining

For Oil Red O (ORO) staining (Sigma-Aldrich, catalog no. O0625), a stock solution was prepared by dissolving powder in 20 ml of 100% isopropanol. The working solution was obtained by adding 3 parts of stock solution to 2 parts of distilled water. After preparing the working solution, the medium was removed from the culture plate and the cells were washed twice with PBS. Then the cells were fixed with 4% paraformaldehyde (PFA) and incubated for 30 min at RT. In the next step, PFA was removed and the cells were washed twice with water, then 60% isopropanol was added and incubated for 5 min. They were then washed 5× with distilled water to remove excess dye. Hematoxylin was subsequently added to the cells and incubated for 1 min. After incubation hematoxylin was discarded and the cells were washed 5× with water. Finally, the cells were covered with water and viewed under a light microscope.

The lipid content in cultured cells was determined using Nile Red staining (Sigma-Aldrich, catalog no.72485), a vital lipophilic and selective fluorescent stain for intracellular lipid droplet accumulation. Staining has been carried out on 4% PFA-fixed cells. Then cells were washed twice with PBS and incubated for 10 min with 3 μM Nile Red solution in PBS. After Nile Red treatment, nuclei were stained by a 5-min incubation in 100 nM solution of 4′,6′-diamidino-2-phenylindole dihydrochloride (DAPI) (Sigma-Aldrich, catalog no. D9542) in PBS. Monolayers were then washed 3× in PBS and used for fluorescence microscopy.

Images were acquired using a Leica TCS SP8 confocal microscope (Leica) with an HCX PL APO ×63/1.40 objective. DAPI fluorescence was measured with excitation of 360/20 nm and emission of 460/20 nm, whereas Nile Red fluorescence was determined using 530/15-nm excitation and 570/15-nm emission. Confocal *z* stacks were acquired with sections of 0.50 μm. Quantification of lipid droplet number and nuclei was done using ImageJ software (Auto Local Threshold tool, Otsu method).

### Immunofluorescence

Whole spheroids were harvested and fixed in 4% PFA with 8% sucrose overnight, then washed 3× in PBS for 1 h. Spheroids were processed for immunofluorescence according to the following conditions: permeabilization and blocking with B-PBT (1% Triton X-100, 10% FBS and 4% BSA in PBS) for 2 h at RT, followed by incubation with primary antibody (Supplementary Table 6) in B-PBT overnight a 4 °C. Then two washes for 2 h in 0.2% PBT (0.2% Triton X-100 in PBS) and one wash in B-PBT for 2 h before incubating in secondary antibody in B-PBT overnight at 4 °C. After two washes in 0.2% PBT and one wash in PBS, cell nuclei were counterstained with DAPI. Images were acquired using a Leica TCS SP8 confocal microscope with HCX PL APO ×40/1.25 objective.

### Isolation of lipid droplets from HepG2 cells

Lipid droplets were isolated from HepG2 cells using the method described previously with minor modifications^71^. Briefly, HepG2 cells treated with oleic acid/palmitic acid 250 µM each for 48 h, and scraped and collected after rinsing with ice-cold PBS. Then cells were resuspended in 10 ml of buffer A (25 mM tricine, 250 mM sucrose, pH 7.8) containing 0.2 mM phenylmethylsulfonyl fluoride and incubated on ice for 20 min. Cells were then homogenized using a Dounce homogenizer in ice, centrifuged at 3,000*g* for 10 min, the postnuclear supernatant fraction was collected and loaded into a SW40 tube and the sample was overlaid with 2 ml of buffer B (20 mM Hepes, 100 mM KCl and 2 mM MgCl_2_, pH 7.4). Samples were centrifuged at 182,000*g* for 1 h at 4 °C. The white (lipid-containing) band containing lipid droplets at the top of gradient was collected into a 1.5-ml Eppendorf tube and washed twice with buffer B. The protein precipitation was carried out using chloroform:acetone (1:3, v/v) treatment followed by centrifugation at 20,000*g* for 10 min at 4 °C. The protein pellet was then dissolved in a 2× sodium dodecylsulfate sample buffer.

### Methylation analysis

The QIAamp circulating nucleic acid kit (QIAGEN) was used for cfDNA extraction, where the vacuum pump was replaced by centrifugation at 8,000*g* for 30 s. From every patient sample, 1 ml of plasma was put through the column (QIAamp Mini-Elute Column) and eluted in 40 μl of elution buffer at the final step. The Enzymatic Conversion Module (New England Biolabs) was used for DNA conversion. For the initial volume of 28 μl, 6.2 μl of 0.2 μg μl^−1^ of carrier RNA (from the QIAmp kit) was combined with 21.8 μl of cfDNA. AMPure XP magnetic beads (Beckman Coulter, Inc.) were used in the clean-up steps, with a magnetic rack (Fisher Scientific UK Ltd). Methprimer^72^ was then used to design primers for converted DNA at the ESR1 site upstream of *PNPLA3*. For the seminested PCR, two pairs of primers per genomic region were designed and the pair containing Illumina partial adapter sequences was used in the second PCR (Supplementary Table 5). Hot Start EpiMark Taq polymerase (New England Biolabs, catalog no. M0490S) was used for seminested PCR following the manufacturer’s instructions, with 1.5 μl of cfDNA as a template for the first PCR and 1 μl of the first PCR mixture as a template for the second PCR. The PCR protocol was 30 s of 95 °C, 30 s of the appropriate *T*_m_ (primer melting temperature) and 45 s of 68 °C. The second PCR was performed for 40 cycles instead of 20 and a different *T*_m_, with but no other changes. The PCR products were visualized via agarose gel electrophoresis, on a 1.5% agarose gel, and the PCR products were either cleaned up with AMPure XP magnetic beads (Thermo Fisher Scientific, catalog no. 10136224) or extracted from the gel using QIAquick Gel Extraction Kit (QIAGEN, catalog no. 28706×4), depending on the presence or absence of primer dimer bands. Quantification of the final DNA product was done with a NanoDrop machine.

Indexed library construction (Illumina 16S amplicon-seq kit) and PE150 sequencing were performed on a Nextseq with approximately 40,000 reads per sample.

The quality of the data was assessed with FastQC and MultiQC^73^ software. The methylation status of each CpG site was extracted using BiQ Analyzer^74^.

For descriptive statistics, as methylation data are not normally distributed, they were converted using the inverse normal transformation technique. The occurrence of possible associations between methylation state and *PNPLA3* genotype was performed by fitting data to bivariate generalized linear models (GLMs).

### Statistical analysis

Analyses were performed using GLMs: linear regression models were fit to analyze continuous traits (aminotransferases), ordinal regression for semiquantitative features of NAFLD-related liver damage (grade of steatosis, ballooning and lobular inflammation, stage of fibrosis), logistic regression for binary traits (NASH and clinically significant fibrosis). Models were adjusted for confounding factors, including study cohort (liver versus severe obesity clinic), age, BMI and the presence of impaired fasting glucose (IFG) or T2D. Genetic associations were tested assuming an additive model. Variables with skewed distributions were logarithmically transformed before entering the models. The interaction between *PNPLA3* p.I148M and other genetic risk variants with sex was tested by entering the interaction term (female sex × number of *PNPLA3* p.I148M risk alleles) in the aforementioned models. Results were reported conforming to the ‘Sex And Gender Equity in Research’ guidelines^75^. In particular, we have: (1) reported the relevance of the study findings for female individuals in both the title and the abstract; (2) focused the study on the evaluation of the sex-specific impact of the *PNPLA3* p.I148M variant on the risk FLD in women (stratified by age and/or menopausal status) versus men; (3) reported all data and genetic associations in clinical cohorts reported specifically in males and females; (4) furthermore, reported results of animal experiments in a sex-specific fashion; and (5) considered in cellular experiments the sex of and hormonal status of cells. Statistical analyses were carried out using the JMP 12.0 (SAS Institute, Cary, NC, USA) and R statistical analysis software v.4.1.1 (http://www.R-project.org). *P* < 0.05 was considered statistically significant.

### Reporting summary

Further information on research design is available in the Nature Portfolio Reporting Summary linked to this article.

## Online content

Any methods, additional references, Nature Portfolio reporting summaries, source data, extended data, supplementary information, acknowledgements, peer review information; details of author contributions and competing interests; and statements of data and code availability are available at 10.1038/s41591-023-02553-8.

### Supplementary information

Supplementary Information Supplementary Tables 1–6 and Figs. 1 and 2.

Reporting Summary

Supplementary Data 1 List of genes differentially expressed in women carrying the *PNPLA3* p.I148M variant.

Supplementary Data Unprocessed files of western blot acquisition.

### Source data

Source Data Fig. 2 Statistical source data.

Source Data Fig. 3 Statistical source data.

Source Data Fig. 3 Unprocessed western blots with relative molecular mass.

Source Data Fig. 4 Statistical source data.

Source Data Fig. 5 Statistical source data.

Source Data Fig. 5 Unprocessed western blots with relative molecular mass.

Source Data Extended Data Fig. 4 Statistical source data.

Source Data Extended Data Fig. 5 Statistical source data.

Source Data Extended Data Fig. 6 Statistical source data.

Source Data Extended Data Fig. 6 Unprocessed western blots with relative molecular mass.

Source Data Extended Data Fig. 3 Statistical source data.

Source Data Extended Data Fig. 4 Unprocessed western blots with relative molecular mass.

## Data Availability

The ethical approval of the study does not allow the public sharing of individual patients’ genetic data. However, anonymized clinical and genetic data that support the findings of the present study are available upon reasonable request to the corresponding author. Sequence data are available upon request. Customized algorithms or software was not used to generate the results reported in this manuscript and is available upon request. All requests for raw and analyzed data and materials will be considered by the corresponding author. Any data and material that can be shared will be released after a material transfer agreement. RNA-seq data that support the findings of the present study have been deposited at the Gene Expression Omnibus under accession no. GSE239422. Please submit a request to corresponding author L.V. (luca.valenti@unimi.it). Source data are provided with this paper.
