# Author Correction: Interaction between estrogen receptor-α and *PNPLA3* p.I148M variant drives fatty liver disease susceptibility in women

**DOI:** 10.1038/s41591-024-02817-x

**Published:** 2024-01-24

**Authors:** Alessandro Cherubini, Mahnoosh Ostadreza, Oveis Jamialahmadi, Serena Pelusi, Eniada Rrapaj, Elia Casirati, Giulia Passignani, Marjan Norouziesfahani, Elena Sinopoli, Guido Baselli, Clara Meda, Paola Dongiovanni, Daniele Dondossola, Neil Youngson, Aikaterini Tourna, Shilpa Chokshi, Elisabetta Bugianesi, EPIDEMIC Study Investigators, Luisa Ronzoni, Cristiana Bianco, Laura Cerami, Veronica Torcianti, Giulia Periti, Sara Margarita, Rossana Carpani, Francesco Malvestiti, Ilaria Marini, Melissa Tomasi, Angela Lombardi, Jessica Rondena, Marco Maggioni, Roberta D’Ambrosio, Valentina Vaira, Anna Ludovica Fracanzani, Chiara Rosso, Grazia Pennisi, Salvatore Petta, Antonio Liguori, Luca Miele, Federica Tavaglione, Umberto Vespasiani-Gentilucci, Marcello Dallio, Alessandro Federico, Giorgio Soardo, Jussi Pihlajamäki, Ville Männistö, Sara Della Torre, Daniele Prati, Stefano Romeo, Luca Valenti

**Affiliations:** 1 Precision Medicine—Biological Resource Center and Department of Transfusion Medicine, Fondazione IRCCS Ca’ Granda Ospedale Maggiore Policlinico, Milan, Italy; 2 Department of Molecular and Clinical Medicine, Gothenburg University, Gothenburg, Sweden; 3 Department of Pathophysiology and Transplantation, Università degli Studi di Milano, Milan, Italy; 4 Department of Health Sciences, Università degli Studi di Milano, Milan, Italy; 5 Medicine and Metabolic Diseases, Fondazione IRCCS Ca’ Granda Ospedale Maggiore Policlinico, Milan, Italy; 6 General and Liver Transplant Surgery, Fondazione IRCCS Ca’ Granda, Ospedale Maggiore Policlinico and University of Milan, Centre of Preclinical Research, Milan, Italy; 7 Foundation for Liver Research, The Roger Williams Institute of Hepatology, London, UK; 8 Faculty of Life Sciences and Medicine, King’s College London, London, UK; 9 Department of Medical Sciences, Division of Gastroenterology, University of Turin, Turin, Italy; 10 Department of Pharmaceutical Sciences, Università degli Studi di Milano, Milan, Italy; 11 Cardiology Department, Sahlgrenska Hospital, Gothenburg, Sweden; 12 Department of Medical and Surgical Science, Magna Græcia University, Catanzaro, Italy; 13 Department of Pathology, Fondazione IRCCS Ca’ Granda Ospedale Maggiore Policlinico, Milan, Italy; 14 Division of Gastroenterology and Hepatology Foundation, IRCCS Ca’ Granda Ospedale Maggiore Policlinico, Milan, Italy; 15 Department of Health Promotion, Mother and Child Care, Internal Medicine and Medical specialties (ProMISE), University of Palermo, Palermo, Italy; 16 Dipartimento Universitario Medicina e Chirurgia Traslazionale, Università Cattolica del Sacro Cuore, Rome, Italy; 17 Area Medicina Interna, Gastroenterologia e Oncologia Medica, Fondazione Policlinico A. Gemelli IRCCS, Rome, Italy; 18 Department of Internal Medicine, Research Unit of Hepatology, Università Campus Bio-Medico di Roma, Rome, Italy; 19 Department of Precision Medicine, University of Campania ‘Luigi Vanvitelli’, Naples, Italy; 20 Clinica Medica, Department of Medicine, European Excellence Center for Arterial Hypertension, University of Udine, Udine, Italy; 21 Department of Clinical Nutrition, Institute of Public Health and Clinical Nutrition, University of Eastern Finland, Kuopio, Finland; 22 Department of Medicine, Endocrinology and Clinical Nutrition, Kuopio University Hospital, Kuopio, Finland; 23 Department of Medicine, University of Eastern Finland and Kuopio, University Hospital, Kuopio, Finland

**Keywords:** Liver diseases, Personalized medicine

Correction to: *Nature Medicine* 10.1038/s41591-023-02553-8, published online 25 September 2023.

In the version of the article initially published, in Table 1, the “Transcriptomic cohort” section inadvertently duplicated data from the “Liver biopsy cohort” section above. The table has been amended to present the correct data in the HTML and PDF versions of the article.

