# Supplementary material for: Interaction between estrogen receptor-α and PNPLA3 p.I148M variant drives fatty liver disease susceptibility in women
Source: Nat Med. 2023 Sep 25;29(10):2643–55. doi: 10.1038/s41591-023-02553-8 (PMC10579099; doi:10.1038/s41591-023-02553-8)

nature medicine

Article https://doi.org/10.1038/s41591-023-02553-8

Interaction between estrogen receptor-α and *PNPLA3* p.I148M variant drives fatty liver disease susceptibility in women

In the format provided by the authors and unedited

Supplementary Tables

	Sex, F		*PNPLA3*, p.I148M alleles		Interaction	
	Estimate ± SE	p value	Estimate ± SE	p value	Estimate ± SE	p value
	Liver Biopsy Cohort (n=1861)					
**Steatosis, grade**	-0.230±0.048	$2*10^{-6}$	+0.640±0.064	$2*10^{-21}$	+0.325±0.063	$2*10^{-7}$
**Ballooning, grade**	-0.200±0.054	0.0002	+0.252±0.070	0.0003	+0.215±0.070	0.002
**Lobular inflammation, grade**	-0.148±0.049	0.0026	+0.398±0.065	$2*10^{-9}$	+0.210±0.064	0.0011
**Fibrosis, stage**	-0.226±0.049	$2*10^{-9}$	+0.433±0.064	$2*10^{-11}$	+0.147±0.063	0.021
**NASH, yes**	-0.151±0.052	0.0035	+0.657±0.070	$9*10^{-21}$	+0.252±0.070	$3*10^{-4}$
**Clinically significant fibrosis, stage >2**	-0.240±0.067	$3*10^{-4}$	+0.544±0.087	$3*10^{-10}$	+0.172±0.084	0.045
**ALT, log IU/l**	-0.154±0.016	$1*10^{-21}$	+0.192±0.021	$4*10^{-19}$	+0.036±0.010	0.049
**AST, log IU/l**	-0.048±0.013	$2*10^{-4}$	+0.157±0.017	$8*10^{-20}$	+0.036±0.017	0.032
	Severe metabolic FLD case-control cohort (n=4374)					
**Severe FLD, yes**	+0.043±0.068	0.53	+0.855±0.067	$9*10^{-23}$	+0.184±0.087	0.034
	Liver-Bible-2022 metabolic dysfunction cohort (n=1142)					
**ALT, log IU/l**	-0.165±0.015	$9*10^{-27}$	+0.097±0.024	$4*10^{-5}$	+0.051±0.024	0.031
	UK Biobank cohort (n=347,127; 35,521 with MRI-PDFF)					
**PDFF, RINT, %***	-0.470±0.011	$<10^{-307}$	0.2±0.011	$2*10^{-49}$	0.037±0.015	0.016
**ALT, RINT IU/l***	-0.637±0.004	$<10^{-307}$	0.1±0.004	$1*10^{-147}$	0.004±0.005	0.451

Supplementary Table 1. Interaction between the *PNPLA3* p.I148M variant and female sex in the pathogenesis of FLD-related liver damage. Upper panel: Impact on histological outcomes and liver enzymes in 1861 individuals at risk in the cross-sectional LBC. NASH: nonalcoholic steatohepatitis. Analyses were performed using multiple generalized linear models adjusted for age, BMI, first 10 principal components of ancestry, and genotyping array. Middle upper panel: Impact on the risk of severe FLD (advanced fibrosis or hepatocellular carcinoma) in 4374 individuals from the severe FLD case-control cohort; the logistic regression model was adjusted for age. Middle lower panel: Impact on ALT levels in 1142 individuals with metabolic dysfunction in the Liver-Bible-2022 cohort; adjusted for ethnicity, age, BMI and T2D. Lower panel: impact on hepatic fat content and ALT levels in 347,127 individuals in the UKBB Biobank population-based cohort. MRI-PDFF: Magnetic resonance imaging proton density fat fraction. RINT: rank-based inverse normal transformation. Analyses were performed using multiple generalized linear models.

	N= Men/Women	Sex, F		*PNPLA3*, p.I148M alleles		Interaction	
		Estimate ± SE	p value	Estimate ± SE	p value	Estimate ± SE	p value
**Liver Biopsy Cohort, Steatosis grade**							
**Fertile, <45 years**	474/379	-0.192±0.035	$6*10^{-8}$	+0.271±0.046	$5*10^{-9}$	+0.104±0.046	0.023
**Transition, 45-55 years**	210/273	-0.060±0.047	0.176	+0.385±0.063	$3*10^{-9}$	+0.123±0.062	0.049
**Menopause, ≥55 years**	257/268	-0.042±0.043	0.335	+0.251±0.065	$1*10^{-5}$	+0.214±0.057	0.0002
**Liver-Bible-2022 Cohort, ALT (log IU/l)**							
**Age <55 years**	495/103	-0.147±0.021	$4*10^{-12}$	+0.076±0.032	0.017	+0.039±0.032	0.22
**Menopause, 55-65 years**	456/88	-0.182±0.022	$1*10^{-15}$	+0.123±0.034	0.0004	+0.068±0.034	0.047
**UK Biobank Cohort, (MRI-PDFF RINT %)**							
**Transition, 45-55 years**	1996/2446	-0.537±0.030	$5*10^{-67}$	+0.163±0.031	$2*10^{-7}$	0.007±0.042	0.86
**Menopause, ≥55 years**	15363/15716	-0.463±0.012	$<10^{-307}$	+0.163±0.012	$7*10^{-44}$	0.042±0.017	0.011
**UK Biobank Cohort, ALT (RINT IU/l)**							
**Fertile, <45 years**	15469/17695	-0.977±0.011	$<10^{-307}$	+0.091±0.01	10^{-16}	-0.055±0.015	0.0002
**Transition, 45-55 years**	42537/53432	-0.820±0.007	$<10^{-307}$	+0.095±0.007	$4*10^{-41}$	-0.015±0.009	0.10
**Menopause, ≥55 years**	102496/115498	-0.490±0.005	$<10^{-307}$	+0.10±0.005	$5*10^{-96}$	0.025±0.007	0.0002

Supplementary Table 2. Interaction between the *PNPLA3* p.I148M variant and female sex in determining fatty liver. Upper panel: impact on steatosis grade in 1861 individuals at risk in the cross-sectional LBC stratified by age class. Analyses were performed at multivariate generalized linear models, adjusted for age, BMI, type 2 diabetes and study sub-cohort; Middle panels: impact on ALT levels (log IU/l) in 817 individuals aged 40-65 with metabolic risk factors for NAFLD in the overall Liver-Bible-2021 cohort. Analyses were performed at multivariate generalized linear models adjusted for ethnicity, age, BMI, type 2 diabetes. The *TM6SF2* p.E167K variant was not significantly associated with ALT in this cohort. Lower panel: UK Biobank cohort, hepatic fat content (n=35,521), MRI-PDFF: Magnetic resonance imaging proton density fat fraction. Analyses were performed using multiple generalized linear models.

	TM6SF2, p.E167K alleles		*MBOAT7*, r641738 T alleles	
	Estimate ± SE	p value	Estimate ± SE	p value
**Liver Biopsy Cohort**				
Steatosis, grade	-0.078±0.114	0.49	-0.113±0.062	0.071
Ballooning, grade	-0.075±0.122	0.54	+0.068±0.071	0.33
Lobular inflammation, grade	-0.030±0.116	0.79	+0.004±0.064	0.94
Fibrosis, stage	-0.104±0.114	0.36	+0.021±0.064	0.75
NASH, yes	-0.030±0.131	0.81	+0.016±0.075	0.83
Clinically significant fibrosis, yes	-0.160±0.146	0.27	-0.060±0.088	0.49
ALT, log IU/l	-0.012±0.043	0.78	+0.001±0.022	0.99
AST, log IU/l	-0.048±0.035	0.17	-0.011±0.018	0.55
**Severe FLD case-control Cohort**				
Severe FLD, yes	+0.047±0.163	0.77	+0.093+0.085	0.27

Supplementary Table 3. Interaction between the *TM6SF2* p.E167K and *MBOAT7* rs641738 variants and female sex in the pathogenesis of FLD-related liver damage. Upper panel: in 1861 individuals at risk in the cross-sectional LBC. NASH: nonalcoholic steatohepatitis. Analyses were performed at multivariate generalized linear models adjusted for age, BMI, type 2 diabetes, study sub-cohort, the genetic variant under evaluation and sex. Bottom: in 4374 individuals from the severe metabolic FLD case-control cohort (adjusted for age, the genetic variant under evaluation and sex). The interaction term (genetic variant * sex) is reported in the table. In this cohort, both rs58542926 *TM6SF2* (p=0.0008) and rs641738 *MBOAT7* (p=0.0007) were associated with severe FLD; rs72613567 *HSD17B13* was also associated with protection against FLD (p<0.0001), but there was no significant interaction with sex as well. Analyses were performed at multivariate generalized linear models adjusted for ethnicity, age, BMI, type 2 diabetes.

	**PNPLA3 mRNA**	
	Estimate ± SE	p value
**Age, years**	-0.005 ± 0.004	0.22
**Sex, F**	+0.171 ± 0.062	0.007
**BMI, Kg/m^2**	+0.011 ± 0.006	0.066
**T2D, yes**	+0.044 ± 0.070	0.53
**ALT, IU/l**	+0.001 ± 0.001	0.53
***PNPLA3* p.I148M, alleles**	+0.226 ± 0.072	0.002

Supplementary Table 4. Independent determinants of hepatic *PNPLA3* mRNA levels at unadjusted multivariate generalized linear model in 125 patients with obesity included in the Transcriptomic cohort. SE: standard error, BMI: body mass index, T2D: type 2 diabetes, ALT: alanine aminotransferases.

Name	Forward Sequence	Reverse Sequence	Application
**PNPLA3**	TCTACAGTGGCCTTATCCCT	GAAAGTTCGTGGACTTGACT	qRT-PCR
**ACTIN**	GGCATCCTCACCCTGAAGTA	GGGGTGTTGAAGGTCTCAAA	qRT-PCR
**FAR1**	TCTCTTTATTGCGGCAGGGA	TGGTACTCAACTTCACCCCA	qRT-PCR
**SPTLC2**	TCACCTCCTGTAGTGGAGCA	GGCAGGCATGTAGTGGAGCA	qRT-PCR
**Human PNPLA3 E1**	CAGGTTGGCAGAGAAGCTGA	GTGCTCGAACTCCAGTGTCT	ChIP PCR
**Human PNPLA3 E2**	GCAACAACGCAGAGAGTAGAC	TCCTGGAGAGAAACGGCTTC	ChIP PCR
**Human PNPLA3 E3**	GCGTCCTCTCCGGTATCC	GAGAGTCCCAGGCTTCGG	ChIP PCR
**PNPLA3-EDIT-ERE-guide1**	CCGGCCTCTTCTCCCTACACCACT	AAACAGTGGTGTAGGGAGAAGAGG	CRISPR/Cas9
**PNPLA3-EDIT-ERE-guide2**	CCGGGAGGTCAGGCTGACACTGTC	AAACGACAGTGTCAGCCTGACCTC	CRISPR/Cas9
**PNPLA3 ESR1 Bis 1st PCR**	GTAATTGGGTGAGGGATGTTAGTT	CCACCATAAATCACAAAAATTAATAATAC	Methylation (outer)
**PNPLA3 ESR1 Bis 2nd PCR**	*TCGTCGGCAGCGTCAGATGTGTATAAGAGACA*G AGGGAGAAGAGGTTAGGTTGATATT	*GTCTCGTGGGCTCGGAGATGTGTATAAGAGACAG* CCACCATAAATCACAAAAATTAATAATAC	Methylation (inner) *Partial Illumina adapters*

Supplementary Table 5. Representative sequence for primers designed to apply in RT-qPCR, ChIP assay and genome editing. List of primers and their sequences used for gene editing. All primers are shown from 5’to3’.

Antigen	Supplier	Catalog no.	RRID	Dilution	
				WB	IF
PNPLA3	Abcam	ab81874	AB_10712485	1:1000	
PLIN2	Abcam	ab52356	AB_2223599	1:1000	
GAPDH	Santa Cruz Biotechnology	Sc-47724	AB_627678	1:1000	
COL1A1	Sigma-Aldrich	HPA011795	AB_1847088		1:100
ERα (D8H8)	Cell Signaling	8644	AB_2617128	1:1000	

Supplementary Table 6. Table of primary antibodies used for western blot and immunofluorescence analysis.

Supplementary Figures

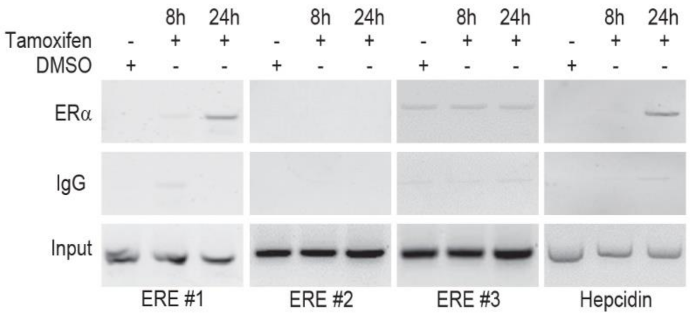

Supplementary Figure 1. Experimental setup of chromatin immunoprecipitation (ChIP) analysis in HepG2 cells. HepG2 cells were treated with tamoxifen (10µM) or DMSO as negative control. The binding of ERα at the ERE1, ERE2 and ERE3 of the PNPLA3 gene or Hepcidin as positive control were tested by PCR and resolved on 2% agarose gel. B) Uncropped version of blots.

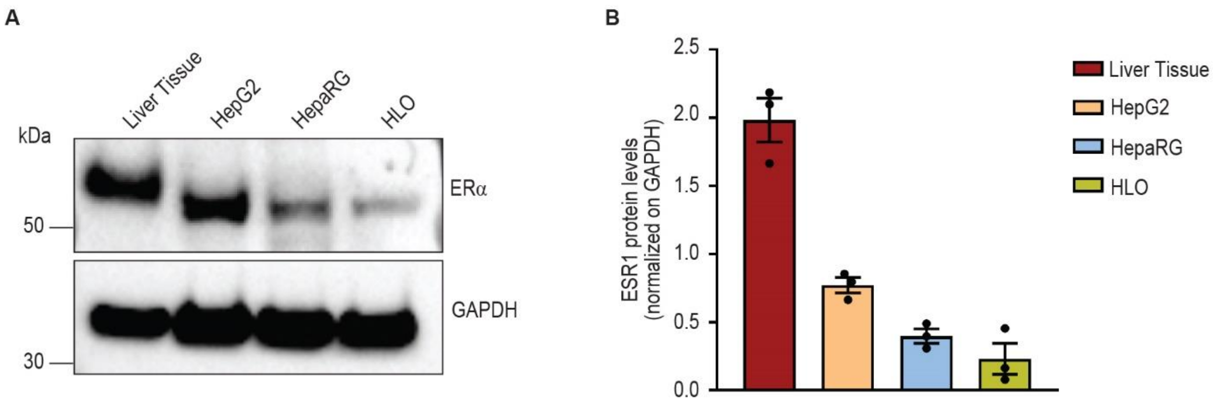

Supplementary Figure 2. Protein levels of estrogen receptor alpha (ESR1) in HepG2, HepaRG and human liver organoids (HLO) compared to human liver tissue. A) Western blot analysis of ESR1 protein levels in human liver tissue, HepG2, HepaRG and HLO (GAPDH used as loading control was run on a different gel) and B) relative quantification. Data in panels B are presented as mean ± SEM (n=3 independent experiments).

Supplementary Data

Supplementary Figure 2A

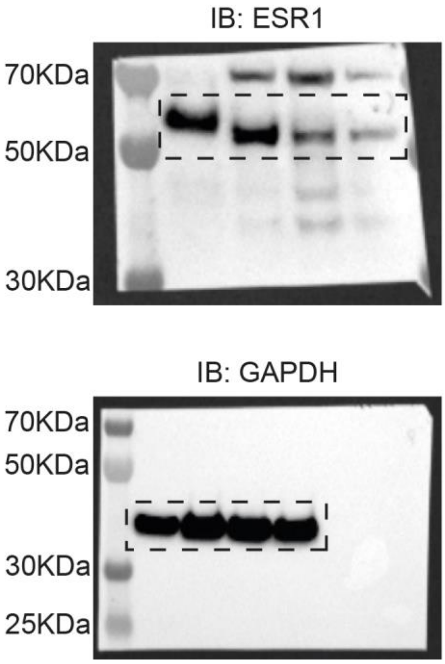

Supplement: Supplementary file 1 — Supplementary Tables 1–6 and Figs. 1 and 2. [file 41591_2023_2553_MOESM1_ESM.pdf]
