# Supplementary material for: Interaction between estrogen receptor-α and PNPLA3 p.I148M variant drives fatty liver disease susceptibility in women
Source: Nat Med. 2023 Sep 25;29(10):2643–55. doi: 10.1038/s41591-023-02553-8 (PMC10579099; doi:10.1038/s41591-023-02553-8)
Supplement: Supplementary file 2 — Reporting Summary [file 41591_2023_2553_MOESM2_ESM.pdf]

nature portfolio

Corresponding author(s): Prof. Luca Valenti

Last updated by author(s): Aug 11, 2023

Reporting Summary

Nature Portfolio wishes to improve the reproducibility of the work that we publish. This form provides structure for consistency and transparency in reporting. For further information on Nature Portfolio policies, see our Editorial Policies and the Editorial Policy Checklist.

Statistics

For all statistical analyses, confirm that the following items are present in the figure legend, table legend, main text, or Methods section.

n/a	Confirmed	
☐	☒	The exact sample size (*n*) for each experimental group/condition, given as a discrete number and unit of measurement
☐	☒	A statement on whether measurements were taken from distinct samples or whether the same sample was measured repeatedly
☐	☒	The statistical test(s) used AND whether they are one- or two-sided<br>*Only common tests should be described solely by name; describe more complex techniques in the Methods section.*
☐	☒	A description of all covariates tested
☐	☒	A description of any assumptions or corrections, such as tests of normality and adjustment for multiple comparisons
☐	☒	A full description of the statistical parameters including central tendency (e.g. means) or other basic estimates (e.g. regression coefficient) AND variation (e.g. standard deviation) or associated estimates of uncertainty (e.g. confidence intervals)
☐	☒	For null hypothesis testing, the test statistic (e.g. *F*, *t*, *r*) with confidence intervals, effect sizes, degrees of freedom and *P* value noted<br>*Give P values as exact values whenever suitable.*
☒	☐	For Bayesian analysis, information on the choice of priors and Markov chain Monte Carlo settings
☒	☐	For hierarchical and complex designs, identification of the appropriate level for tests and full reporting of outcomes
☒	☐	Estimates of effect sizes (e.g. Cohen's *d*, Pearson's *r*), indicating how they were calculated

Our web collection on statistics for biologists contains articles on many of the points above.

Software and code

Policy information about availability of computer code

Data collection	ABI 7500 fast thermo cycler (ThermoFisher Scientific); ChemiDoc system (BioRad); Leica TCS SP8 confocal microscope (Leica, Wetzlar, Germany) with HCX PL APO x63/1.40 objective.
Data analysis	Statistical analyses were carried out using the JMP 12.0 (SAS Institute, Cary, NC, USA), R statistical analysis software version 4.1.1 (http://www.R-project.org/) and GraphPad Prism 8.0.1. To identify differentially expressed pathways, we used p≤0.05 as a cutoff for DEG inclusion criteria for ingenuity pathway analysis (IPA, Qiagen, www.qiagen.com/ingenuity) and gene set enrichment analysis (GSEA, http://www.broad.mit.edu/gsea/). GSEA was performed in the pre-ranked mode, with the dataset (version 7.4), in which phenotypes were permuted 1,000 times to obtain stable analysis results. The promoter region of PNPLA3 gene was analyzed by using Dragon ERE Finder 6.0 60 algorithm to identify the presence of putative estrogen response element (ERE). Quality of the methylation data was assessed with FastQC (v0.11.5) and MultiQC (v1.12). The methylation status of each CpG site was extracted with BiQ Analyzer. Western blot and immunostaining wer quantified using ImageJ 1.53t. RNA sequencing was exploited in paired-end mode with a read length of 150nt using the Illumina HiSeq 4000 (Novogene). Raw reads were aligned on the GRCh37 reference genome using STAR mapper (v2.7.10b). Reads count, according to ENSEMBL human transcript reference assembly version 75, was performed using RSEM package (v1.2.31). Count normalization and differential gene expression analysis were performed using DESeq2 package (1.40.2).

For manuscripts utilizing custom algorithms or software that are central to the research but not yet described in published literature, software must be made available to editors and reviewers. We strongly encourage code deposition in a community repository (e.g. GitHub). See the Nature Portfolio guidelines for submitting code & software for further information.

Data

Policy information about availability of data

All manuscripts must include a data availability statement. This statement should provide the following information, where applicable:

- Accession codes, unique identifiers, or web links for publicly available datasets
- A description of any restrictions on data availability
- For clinical datasets or third party data, please ensure that the statement adheres to our policy

RNA sequencing data that support the findings of this study have been deposited at the Gene Expression Omnibus (GEO) under accession number GSE239422.

Human research participants

Policy information about studies involving human research participants and Sex and Gender in Research.

Reporting on sex and gender	Our manuscript is compliant to the "Sex And Gender Equity in Research" (SAGER) guidelines (PMID: 29451543). Our study consider both sexes with human participants who self-reported their biological sex on the requisition form upon enrollment. Our manuscript report the multiplicative interaction between female sex and PNPLA3 p.I148M variant in the NAFLD outcomes. All data were collected and reported disaggregated for sex.
Population characteristics	The clinical feature of individuals included in the study cohort are reported in Table S3.
Recruitment	For the Liver Biopsy Cohort (LBC) up to July 1st 2018 a total of 1861 individuals were enrolled in four European centers: Milan, Palermo, Rome (Italy), and Kuopio (Finland). The inclusion criteria were liver biopsy for suspected NASH or severe obesity (at the time of initial diagnosis) and availability of DNA samples, as well as clinical data. All subjects were of European descent and were consecutively enrolled at their centers of reference.
Ethics oversight	Informed written consent was obtained from each patient and the study protocol was approved by the Ethical Committee of the Fondazione IRCSS Ca' Granda and the other involved Institutions and conformed to the ethical guidelines of the 1975 Declaration of Helsinki.

Note that full information on the approval of the study protocol must also be provided in the manuscript.

Field-specific reporting

Please select the one below that is the best fit for your research. If you are not sure, read the appropriate sections before making your selection.

☒ Life sciences ☐ Behavioural & social sciences ☐ Ecological, evolutionary & environmental sciences

For a reference copy of the document with all sections, see nature.com/documents/nr-reporting-summary-flat.pdf

Life sciences study design

All studies must disclose on these points even when the disclosure is negative.

Sample size	For the Discovery cohort study we included 1861 individual in Liver Biopsy Cohort (LBC), where we previously demonstrated that the PNPLA3 p.I148M variant and sex were significantly associated with the whole spectrum of histological damage typical of fatty liver disease. Results were replicated in the UK Biobank cohort, a large population-based cohort comprising >350,000 participants where liver enzymes and other indices of liver damage are available.<br>For in vitro study no statistical methods were used to calculate sample size and group size were estimated on the basis of our experience and previous study variance in the field.
Data exclusions	Other causes of liver disease were excluded, including increased alcohol intake (men, >30 g/day; women, >20 g/day), viral and autoimmune hepatitis, hereditary hemochromatosis, alpha1-antitrypsin deficiency, and history infection with hepatitis B or hepatitis C. Patients who had decompensated cirrhosis or were taking drugs that induce steatosis were excluded.
Replication	For the cohort study we succesfully replicated the association between female sex and PNPLA3 p.148M variant in NAFLD outcomes in three independent cohort: fatty liver disease (FLD) cohort composed by 4374 individuals , Liver-Bible-2022 cohort composed by 1142 individuals and UK Biobank cohort (UKBB) database composed by ≈450,000 individuals.<br>For in vitro study all attempts at replication were successful. Each result described in the paper is based on at least three indipendent biological replicates. For human organoids, three different lines were used and all attempts at replication were successful. Representative images from western blot and immunostaining were demostrated.
Randomization	No specific method of randomization was used.
Blinding	This is an observational genetic study, not a clinical trial. For in vitro study to maximez the objectivity, different individuals performed the same experiment.

Reporting for specific materials, systems and methods

We require information from authors about some types of materials, experimental systems and methods used in many studies. Here, indicate whether each material, system or method listed is relevant to your study. If you are not sure if a list item applies to your research, read the appropriate section before selecting a response.

Materials & experimental systems

n/a	Involved in the study
☐	☒ Antibodies
☐	☒ Eukaryotic cell lines
☒	☐ Palaeontology and archaeology
☐	☒ Animals and other organisms
☒	☐ Clinical data
☒	☐ Dual use research of concern

Methods

n/a	Involved in the study
☒	☐ ChIP-seq
☒	☐ Flow cytometry
☒	☐ MRI-based neuroimaging

Antibodies

Antibodies used

Western Blot PNPLA3 Abcam a#b81874 RRID:AB_10712485 1:1000
Western Blot PLIN2 Abcam #ab52356 RRID:AB_2223599 1:1000
Western Blot GAPDH (0411) Santa Cruz Biotechnology #sc-47724 ARRID:B_627678 1:1000
Immunostaining COL1A1 Sigma-Aldrich #HPA011795 RRID:AB_1847088 1:100
Immunostaining Goat anti-Rabbit IgG (H+L) Highly Cross-Adsorbed Secondary Antibody, Alexa Fluor™ Plus 647 ThermoFisher scientific #A32733 1:250
Immunostaining 4′, 6′-diamidino-2-phenylindole dihydrochloride (DAPI) Sigma Aldrich #D9542
Chromatin Immunoprecipitation ERalpha(D8H8) Cell Signaling #8644 AB_2617128

Validation

The antibodies were validate by either western blotting or immunostaining by vendors or previously published papers. Please refer to the vendor website as listed after each antibody below for the detailed validation conditions and results:

- Anti-PNPLA3 antibody (ab81874) https://www.abcam.com/products/primary-antibodies/pnpla3-antibody-ab81874.html
- Rabbit polyclonal to Perilipin-2 (ab52356) https://www.abcam.com/products/primary-antibodies/rabbit-polyclonal-to-perilipin-2-ab52356.html
- Antibody GAPDH (0411): sc-47724 https://www.scbt.com/it/p/gapdh-antibody-0411
- Anti-COL1A1 antibody produced in rabbit https://www.sigmaaldrich.com/IT/it/product/sigma/hpa011795
- Goat anti-Rabbit IgG (H+L) Highly Cross-Adsorbed Secondary Antibody, Alexa Fluor™ Plus 647 https://www.thermofisher.com/antibody/product/Goat-anti-Rabbit-IgG-H-L-Highly-Cross-Adsorbed-Secondary-Antibody-Polyclonal/A32733
- 4′, 6′-diamidino-2-phenylindole dihydrochloride (DAPI) https://www.sigmaaldrich.com/IT/it/product/sigma/d9542

Eukaryotic cell lines

Policy information about cell lines and Sex and Gender in Research

Cell line source(s)

HepG2 (ATCC #HB-8065, RRID:CVCL_0027), HepaRG (ThermoFisher Scientific #HPRGC10, RRID:CVCL_9720), 293T (ATCC #CRL-3216, RRID:CVCL_0063), LX-2 (Millipore #SCC064, RRID:CVCL_5793).

Authentication

Authentications were performed by the company prior to shipping. Cell line identity were confirmed by morphology and immunostaining.

Mycoplasma contamination

All cells were routinely tested to exclude mycoplasma contamination MycoAlertTM PLUS Mycoplasma Detection Kit (Lonza, #LT07-710).

Commonly misidentified lines
(See ICLAC register)

No commonly misidentified cell lines were used.

Animals and other research organisms

Policy information about studies involving animals; ARRIVE guidelines recommended for reporting animal research, and Sex and Gender in Research

Laboratory animals

ERa floxed mice, strain C57Bl/6J, age 8 months.

Wild animals

No wild animals were used in this study.

Reporting on sex

both male and female mice were analyzed; females were collected in two phases of the estrous cycle (proestrus and metestrus, characterized by high and low estrogen levels) after vaginal smear analysis.

Field-collected samples	No field-collected samples were used in this study
Ethics oversight	All animal experimentation was done in accordance with the ARRIVE and European guidelines for animal care and use of experimental animals. The animal study protocol was approved by "Istituto Superiore di Sanità-Ministero della Salute Italiano" (protocol code 1272/2015-PR, date of approval 15 December 2015).

Note that full information on the approval of the study protocol must also be provided in the manuscript.
