# Supplementary figures and images for: Interaction between estrogen receptor-α and PNPLA3 p.I148M variant drives fatty liver disease susceptibility in women
Source: Nat Med. 2023 Sep 25;29(10):2643–55. doi: 10.1038/s41591-023-02553-8 (PMC10579099; doi:10.1038/s41591-023-02553-8)

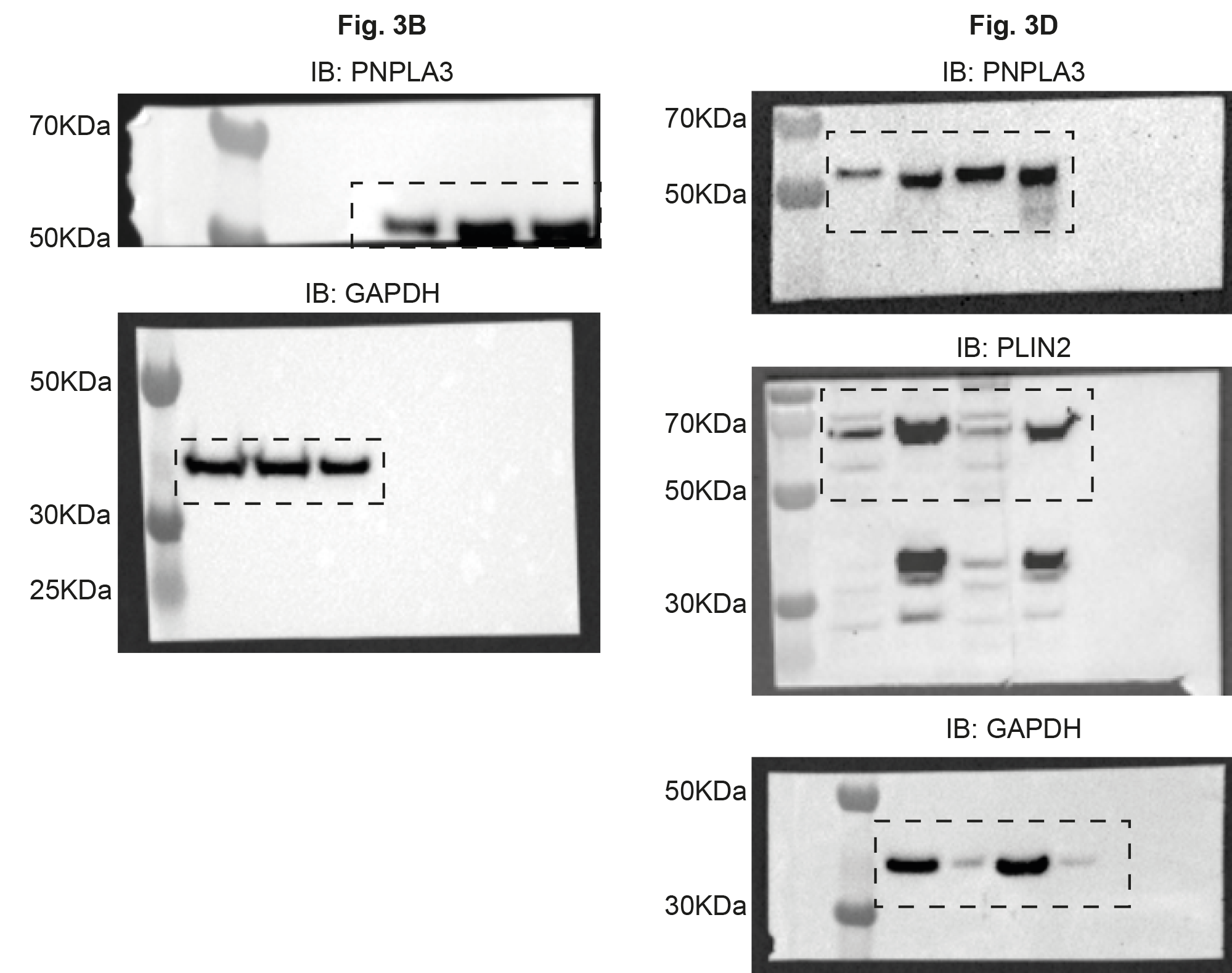

Supplement: Supplementary file 7 — Unprocessed western blots with relative molecular mass. [file 41591_2023_2553_MOESM7_ESM.tif]

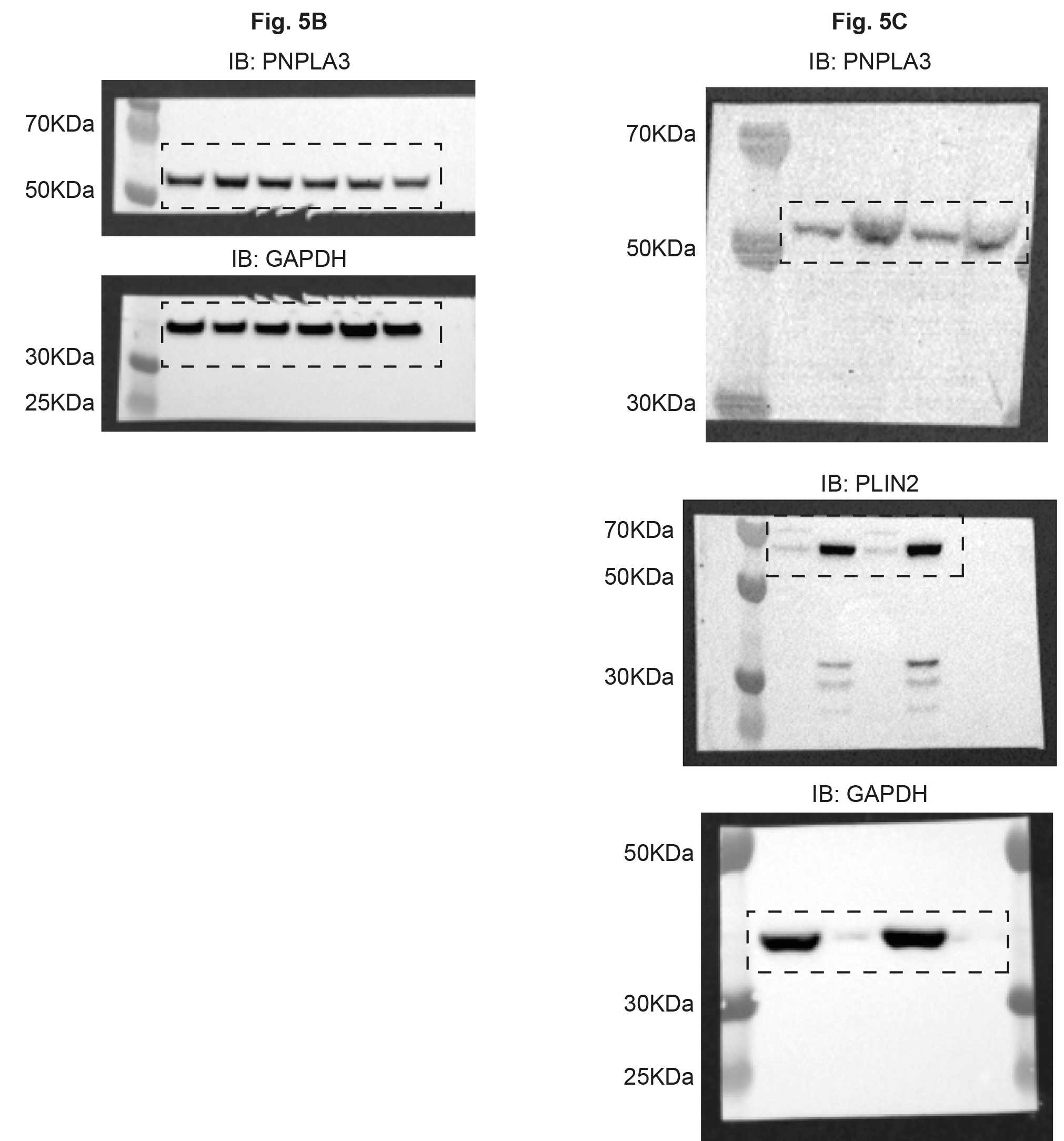

Supplement: Supplementary file 10 — Unprocessed western blots with relative molecular mass. [file 41591_2023_2553_MOESM10_ESM.tif]

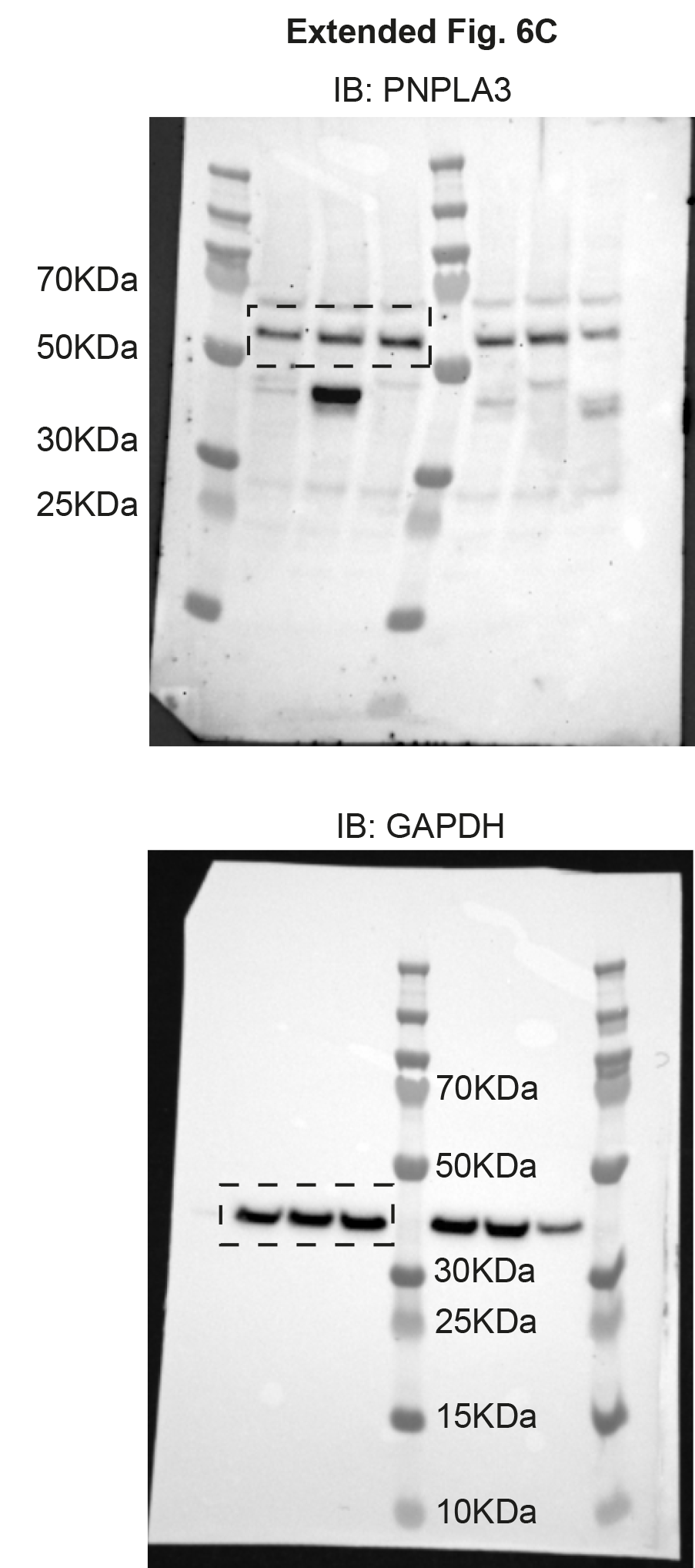

Supplement: Supplementary file 14 — Unprocessed western blots with relative molecular mass. [file 41591_2023_2553_MOESM14_ESM.tif]

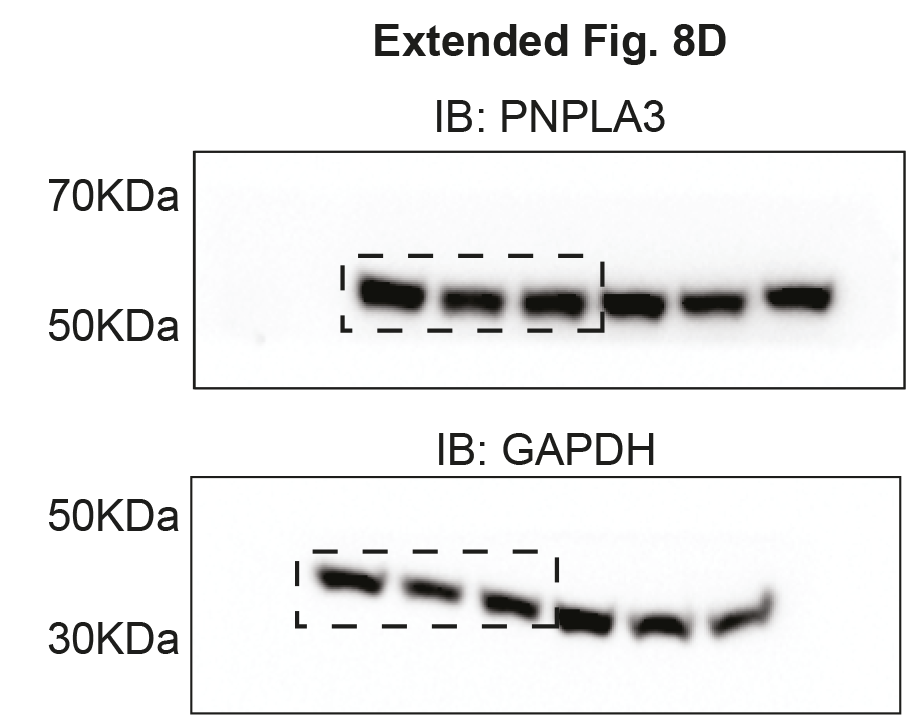

Supplement: Supplementary file 16 — Unprocessed western blots with relative molecular mass. [file 41591_2023_2553_MOESM16_ESM.tif]
